# Tri-trophic consequences of plant-to-plant volatile signalling and its contingency on plant relatedness in wild cotton

**DOI:** 10.1093/aob/mcaf315

**Published:** 2025-12-08

**Authors:** Sandra Díaz-Cruz, Ted C J Turlings, Uriel Solís-Rodríguez, Jonathan Interian-Aguiñaga, Víctor Hugo Ramírez-Delgado, Mary V Clancy, Marine Mamin, Jonathan F Wendel, Corrinne E Grover, Mark A Arick, Chuan-Yu Hsu, Olga Pechanova, Adam Thrash, Daniel G Peterson, Carlos Bustos-Segura, Luis Abdala-Roberts

**Affiliations:** Departamento de Ecología Tropical, Campus de Ciencias Biológicas y Agropecuarias, Universidad Autónoma de Yucatán, Apartado Postal 4-116, Itzimná 97000, Mexico; State Key Laboratory of Crop Stress Adaptation and Improvement, State Key Laboratory of Cotton Bio-Breeding and Integrated Utilization, School of Life Sciences, College of Agriculture, Henan University, Kaifeng 475004, China; Laboratory of Fundamental and Applied Research in Chemical Ecology (FARCE), Institute of Biology, University of Neuchâtel, Rue Emile-Argand 11, Neuchâtel 2000, Switzerland; Department of Entomology, The Pennsylvania State University, University Park, PA 16802, USA; Universidad Nacional Autónoma de México, Escuela Nacional de Estudios Superiores Unidad Mérida, Carretera Mérida-Tetiz Km. 4.5, Ucú, Yucatán 97357, Mexico; Departamento de Ecología Tropical, Campus de Ciencias Biológicas y Agropecuarias, Universidad Autónoma de Yucatán, Apartado Postal 4-116, Itzimná 97000, Mexico; Departamento de Ecología Tropical, Campus de Ciencias Biológicas y Agropecuarias, Universidad Autónoma de Yucatán, Apartado Postal 4-116, Itzimná 97000, Mexico; Laboratory of Fundamental and Applied Research in Chemical Ecology (FARCE), Institute of Biology, University of Neuchâtel, Rue Emile-Argand 11, Neuchâtel 2000, Switzerland; Laboratory of Fundamental and Applied Research in Chemical Ecology (FARCE), Institute of Biology, University of Neuchâtel, Rue Emile-Argand 11, Neuchâtel 2000, Switzerland; Department of Ecology, Evolution, & Organismal Biology, Iowa State University, Ames, IA 50011, USA; Department of Ecology, Evolution, & Organismal Biology, Iowa State University, Ames, IA 50011, USA; Institute for Genomics, Biocomputing & Biotechnology, Mississippi State University, Starkville, MS 39762, USA; Institute for Genomics, Biocomputing & Biotechnology, Mississippi State University, Starkville, MS 39762, USA; Institute for Genomics, Biocomputing & Biotechnology, Mississippi State University, Starkville, MS 39762, USA; Institute for Genomics, Biocomputing & Biotechnology, Mississippi State University, Starkville, MS 39762, USA; Institute for Genomics, Biocomputing & Biotechnology, Mississippi State University, Starkville, MS 39762, USA; Laboratory of Fundamental and Applied Research in Chemical Ecology (FARCE), Institute of Biology, University of Neuchâtel, Rue Emile-Argand 11, Neuchâtel 2000, Switzerland; Institute of Ecology and Environmental Sciences-Paris, INRAE, Sorbonne Université, CNRS, IRD, Université de Paris, UPEC, Route de St Cyr, Versailles 78026, France; Universidad Nacional Autónoma de México, Escuela Nacional de Estudios Superiores Unidad Mérida, Carretera Mérida-Tetiz Km. 4.5, Ucú, Yucatán 97357, Mexico

**Keywords:** Ant–plant interaction, extrafloral nectar, *Gossypium hirsutum* L, indirect defences, predation, volatile organic compounds

## Abstract

**Background and Aims:**

Plant volatile organic compounds (VOCs) induced by herbivory boost defences in neighbouring plants. These effects have been shown primarily for direct plant defences and are often stronger when emitter and receiver plants are genetically related. However, we know much less about how plant indirect defence is affected by VOC signalling. To address this, we conducted field experiments controlling for plant relatedness, testing the effects of VOC signalling on extrafloral nectar (EFN) production, a key indirect defence, and its impact on ant recruitment and attacks on herbivores of wild cotton (*Gossypium hirsutum*) plants.

**Methods:**

Experiments consisted of plant triplets, in which one individual acted as an emitter of VOCs and two as receivers. One receiver shared the same mother plant as the emitter, and the other was descended from a different mother. Half of the emitter plants were induced using the specialist caterpillar *Alabama argillacea*, and VOCs were collected. We then induced receivers and measured their EFN production, in addition to ant abundance and attack on sentinel caterpillars. We also subsequently excluded ants from half of the receivers to test for ant-mediated effects on natural herbivory occurring over the following weeks.

**Key Results:**

Receivers exposed to VOCs of damaged emitters produced a greater volume and concentration of EFN in response to herbivory relative to those exposed to undamaged emitters and, accordingly, showed higher rates of ant attack on sentinel caterpillars (albeit no differences in ant abundance). These effects were not contingent on emitter–receiver relatedness. In addition, we found no effect of ant exclusion on natural herbivory levels on receiver plants, although damage was low overall.

**Conclusions:**

These findings provide insight into inter-plant VOC signalling effects on multitrophic interactions by revealing indirect defence induction that leads to herbivore reduction by ants but that such effect occurs independently of the degree of emitter–receiver relatedness.

## INTRODUCTION

Plants have evolved a diverse array of defences against herbivores, which can be direct defences (i.e. traits that directly deter or harm herbivores, such as toxins and physical barriers) or indirect defences (i.e. traits that attract natural enemies of herbivores, such as extrafloral nectar, EFN, or inform the presence of herbivores, such as volatile organic compounds, VOCs) (War *et al.*, 2012). In particular, plants respond to different types of cues in their environment, among which VOCs have been especially well studied (Karban, 2021; Ninkovic *et al.*, 2021). Notably, plants have been shown to respond to herbivore-induced VOCs emitted by neighbouring plants, which results in the induction or priming (preparing) of their defences, leading to increased resistance to herbivory (Turlings and Erb, 2018; Michereff *et al.*, 2021; Ninkovic *et al.*, 2021). Thus far, these signalling effects have been studied mainly for plant direct defences (Moreira and Abdala-Roberts, 2019), whereas indirect defence responses have been far less documented (but see Kost and Heil, 2006; Heil and Silva-Bueno, 2007; Aljbory and Chen, 2018; Briones-May *et al.*, 2023). As a result, there is currently a poor understanding of the consequences of VOC-mediated inter-plant signalling for higher trophic levels. Accordingly, studying plant–plant signalling effects on indirect defences can fill a key research gap that builds on classic above-ground plant–plant signalling work involving mainly herbivores (or plant pathogens) to link plant volatile-mediated effects to multitrophic interactions and plant-associated food webs, ultimately yielding a more realistic and holistic view of plant signalling embedded within complex arthropod communities.

Extrafloral nectaries are a prominent and widespread indirect defensive trait in plants (present in >100 plant families; Weber and Kheeler, 2013) that attract predators and parasitoids of herbivorous insects (Heil, 2015). Numerous studies have shown that EFN is a sugary solution that provides an important nutritional resource for predators, particularly for ants (Turlings and Wäckers, 2004; Rico-Gray and Oliveira, 2007; Heil, 2015). Moreover, there are strong linkages between EFN induction and plant-associated multitrophic communities composed not only of omnivorous predators but also of other trophic levels and guilds recruited to this food source and triggering direct and indirect effects across trophic levels that can shape arthropod diversity and trophic structure (Marazzi *et al.*, 2013; Heil, 2015). To date, however, only a few studies have examined the effects of VOC-mediated signalling on EFN, including work on lima bean (*Phaseolus lunatus*; Kost and Heil, 2006; Heil and Silva-Bueno, 2007), two *Acacia* species (Hernández-Zepeda *et al.*, 2018), and wild cotton (*Gossypium hirsutum*; Briones-May *et al.*, 2023). Although these studies report on the effects of VOC-mediated signalling on plant resistance, only Kost and Heil (2006) measured ant and predator (wasp) activity. More research is needed testing for inter-plant VOC signalling effects on predator responses to place these dynamics within their food web context and better understand the implications for plant indirect defence and trophic control. Efforts to understand the impacts of these plant signalling effects on EFN induction promise to unlock key knowledge on the importance and mechanisms by which plant volatile-mediated interactions impact associated multitrophic communities and how these dynamics play out to impact plant fitness.

Plant-to-plant signalling is highly context dependent as a function of multiple biotic and abiotic factors (Moreira and Abdala-Roberts, 2019; Karban, 2021). In this sense, the degree of relatedness between emitter and receiver plants can be an important source of variation, whereby higher relatedness has been found to lead to stronger signalling effects on defence induction or priming, and resistance to herbivory in receivers (reviewed by Moreira and Abdala-Roberts, 2019; Karban, 2021). The effect of emitter–receiver relatedness has been tested in several ways, including the use of emitters and receivers of the same vs. different populations (e.g. *P. lunatus*: Moreira *et al.*, 2016), different varieties of crop plants (e.g. *Solanum tuberosum*: Martín-Cacheda *et al.*, 2023), and plants of the same clone vs. a different clone (e.g. *Artemisia tridentata*: Karban *et al.*, 2013). Furthermore, one of these studies found evidence of genetically based sex differences in signalling for the dioecious *Baccharis salicifolia* but no effect of plant clone (Moreira *et al.*, 2018), suggesting that different pools of genetically based variation underlie signalling effects in this species. The likely proximal explanation of these findings is quantitative or qualitative similarity in volatile cues among related individuals, as evidenced by chemotypic studies (e.g. Karban *et al.*, 2013, 2024), whereas the ultimate drivers proposed are mechanisms such as kin selection (Karban *et al.*, 2013) or patch-level benefits of heightened resistance (the mutual benefit hypothesis; Kalske *et al.*, 2019; Grof-Tisza *et al.*, 2021; Kessler *et al.*, 2023; Karban *et al.*, 2024). However, little information exists on whether this source of signalling specificity extends to or involves indirect defence and plant-associated multitrophic interactions.

**Fig. 1.**
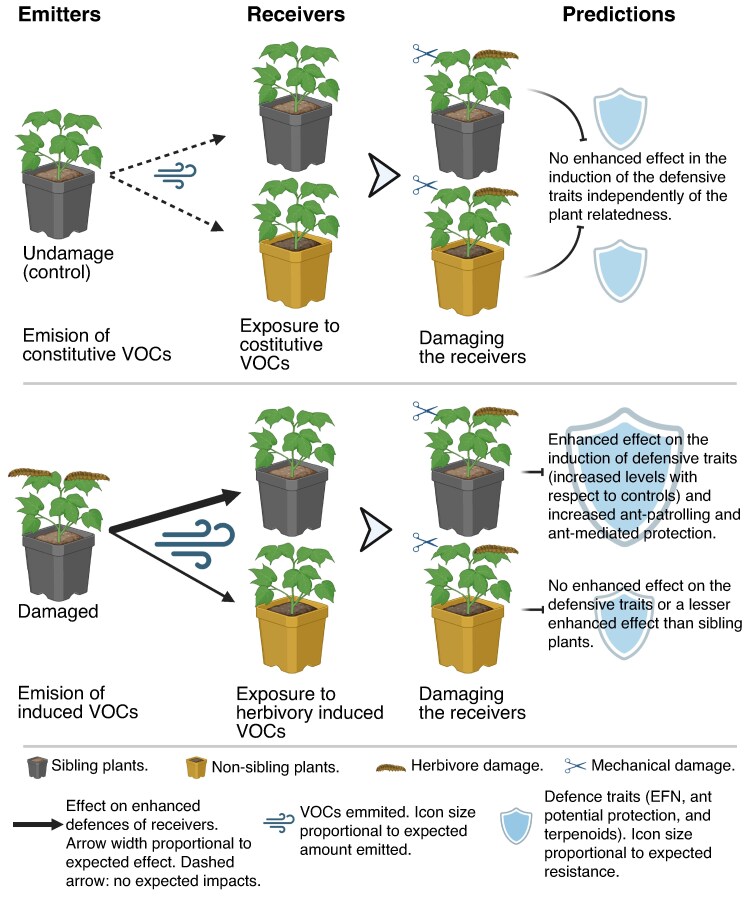
Conceptual design and predictions of our experiments. Triplets of wild cotton plants consisting of two siblings and one non-sibling acted as our experimental units. One sibling plant acted as the emitter, while the other two plants were the receivers. We show the expected outcomes on receiver defensive responses [extrafloral nectar (EFN); ant patrolling and attack on herbivores; and terpenoid compounds] when exposed to volatile organic compounds (VOCs) from either damaged or undamaged emitters and their contingency on relatedness (sibling or non-sibling plants). We predicted that damaged emitters would enhance the induction of indirect defensive traits in receivers, with stronger effects when the emitter and receiver were more closely related. See Materials and Methods for experimental details. Created in BioRender. Delgado, V. (https://BioRender.com/rpwa0zp) is licensed under CC BY 4.0.

Cotton (*Gossypium hirsutum*) is an EFN-producing myrmecophyte (i.e. it establishes mutualistic interactions with ants) found in the coastal shrubland of northern Yucatan (Mexico). The induction of EFN (Quijano-Medina *et al.*, 2021; Vázquez-Barrios *et al*., 2021; Briones-May *et al.*, 2023) and VOCs (Hagenbucher *et al.*, 2013; Grandi *et al.*, 2024; Mamin *et al.*, 2025; Martín-Cacheda *et al.*, 2025) in response to herbivory has been well studied in this species. Herbivory on cotton induces both direct chemical defences (e.g. phenolic compounds, terpenoid aldehydes; reviewed by Hagenbucher *et al.*, 2013) and indirect defences, such as EFN (Quijano-Medina *et al.*, 2021; Briones-May *et al.*, 2023) and VOCs that might attract natural enemies of herbivores (McCall *et al.*, 1993; Röse *et al.*, 1998; Mobarak *et al.*, 2025). Furthermore, VOCs induced by herbivory also mediate inter-plant signalling (Bruin *et al.*, 1992; Grandi *et al.*, 2024; Quijano-Medina *et al.*, 2024; Mamin *et al.*, 2025) and affect chemical defences and EFN production in exposed plants (Briones-May *et al.*, 2023; Grandi *et al.*, 2024), thereby contributing to plant resistance to herbivory (e.g. Zakir *et al.*, 2013; Grandi *et al.*, 2024; Martín-Cacheda *et al.*, 2025). Much of the earlier work on VOCs and signalling centred on cultivated cotton (reviewed by Hagenbucher *et al.*, 2013), but studies with wild populations have increased in recent years (e.g. Abdala-Roberts *et al.*, 2019*b*; Quijano-Medina *et al.*, 2021; Clancy *et al.*, 2023; Mamin *et al.*, 2025). Despite this, it remains unknown whether these signalling effects are correlated with emitter–receiver relatedness or have implications for ant–plant interactions and resulting top-down control on wild cotton. To test this, we conducted *in situ* experiments in the coastal shrubland of Yucatan (Mexico). For emitter induction, we used the specialist caterpillar *Alabama argillacea*, and to manipulate emitter–receiver relatedness we used emitter and receiver plants sourced from the same source mother plant or a different one. We collected emitter VOCs and measured receiver EFN production, ant recruitment and anti-herbivore responses (probability of attack on sentinel caterpillars pinned to the plants to measure attack on potential prey), as well as natural herbivory to assess effects on plant resistance (Fig. 1). In doing so, this study provides new mechanistic insight into how inter-plant VOC signalling influences EFN-associated ant recruitment and its consequences for plant indirect defence.

## MATERIALS AND METHODS

### Study species

*Gossypium hirsutum* L. (Malvaceae) is a perennial shrub species that is naturally distributed in Central America, Mexico and the Caribbean Basin (Coppens d’Eeckenbrugge and Lacape, 2014; Wendel and Grover, 2015). It is especially abundant at coastal sites along the northern coast of the Yucatan Peninsula (Mexico), characterized by sandy saline soils with low and highly seasonal rainfall and elevated (in some cases, extreme) temperatures (Coppens d’Eeckenbrugge and Lacape, 2014; Abdala-Roberts *et al.*, 2019*a*; Quijano-Medina *et al.*, 2021). In this region, wild cotton is attacked mainly by leaf-chewing insects (e.g. caterpillars and grasshoppers; Abdala-Roberts *et al.*, 2019*a*), among which the specialist *Alabama argillacea* Hübner, 1823 (Lepidoptera: Erebidae) is one of the most common (L. Abdala-Roberts, personal observation). Larvae feed mainly on stems and young leaves (Quirino and Soares, 2001), and in some regions this insect is considered an important pest of cultivated cotton (Helman *et al.*, 2011; Nascimento *et al.*, 2011). At coastal sites in Yucatan, this insect is found throughout the distribution range of wild cotton and can reach outbreak levels at some sites (L. Abdala-Roberts, personal observation).

### Plant material

In 2023, we collected seeds from nine adult cotton plants (mothers) found across several adjacent sites located near the coastal town of Celestún, Yucatán, México (20°59'53.9"N, 90°20'13.7"W). Distances between sites were 1–2 km, and seeds from the same plant are referred to hereafter as siblings (vs. non-siblings, i.e. plants from a different mother plant). Plants from the same mother plant were presumably a mix between full- and half-sib plants, because this species has a mixed mating system (Wendel *et al.*, 1992; Brubaker and Wendel, 1994; Velázquez-López *et al.*, 2018). Seeds exhibit low dispersal and, in most cases, fall within the vicinity of mother plants (L. Abdala-Roberts, personal observation). Outcrossing rates (i.e. the proportion of offspring produced through cross-pollination within cotton populations) have been shown to be moderate to high (Velázquez-López *et al.*, 2018) which could weaken spatial structuring into genetic families within populations (Loveless and Hamrick, 1984; Linhart and Grant, 1996). The relative strength of these opposing forces within (and among) populations will thus determine the extent to which VOC-mediated plant–plant interactions could be shapped by genetic relatedness (i.e. within and among families; Karban, 2021), particularly in less-studied species with mixed mating systems, such as wild cotton. Accordingly, we sampled leaves to determine genetic relatedness within and among sibling cohorts by calculating the genetic distance for a subsample of seven to 10 offspring from each mother plant (Supplementary Data Fig. S1). The methods used to process and analyse these samples and obtain genetic distances are described in the Supplementary Data (Section S1), and the main findings are reported in the Results section.

### Experimental procedures

In late April 2023, seeds were germinated in Petri dishes with wetted cotton at 35 °C for 48 h. Germinated seeds were planted in plastic trays with a mixture of agrolite, sand (from the seed source sites) and tropical forest substrate (1:1:2), and kept for 3 weeks in a greenhouse at the Campus de Ciencias Biológicas y Agropecuarias (CCBA) of the Universidad Autónoma de Yucatán (20.8657427N, −89.6222614W). Three-week-old seedlings were taken to a greenhouse at the Unidad Multidisciplinaria de Docencia e Investigación of the Universidad Nacional Autónoma de México (21.1633785N, −90.0488892W) located at the coastal town of Sisal, Yucatan. Seedlings were transplanted to 2 L plastic bags with the same soil mix, where they were kept for two months and watered with 300 mL every three days. Two-and-a-half-month-old plants (measuring 30–40 cm in height) were transported to a nearby coastal shrubland site (21.1967818N, −89.95614117W), where the experiments took place.

### Experiment 1: effects of signalling and plant relatedness on ant recruitment and herbivory resistance

We established 72 experimental units at the field site, where each one was composed of three potted plants (*n* = 216 plants). For each unit, one plant acted as a VOC emitter and the other two as receivers. To evaluate relatedness effects, one of the receivers was a sibling and the other a non-sibling of the emitter plant. Spacing between triplets was ≥1.5 m, and the distance between plants within each unit was 20 cm (preventing physical contact between plants).

To test VOC-mediated signalling effects attributable to herbivory, we exposed half of the emitter plants to damage by third- or fourth-instar *A. argillacea* caterpillars, whereas the other half were left undamaged. For this, we placed one caterpillar inside a clip cage (6 cm in diameter) on each of two apical leaves per plant. Control emitters also had clip cages but without caterpillars. Caterpillars were left on plants for 72 h, changing the position of the clip cage each day as accessible leaf tissue within the clip cage was consumed by caterpillars. Mother plants were represented similarly across herbivory treatment levels and herbivory–relatedness combinations. We collected emitter VOCs at 48 and 72 h after damage onset for a subsample of 45 plants (22 controls and 23 damaged). The day after collecting VOCs, we removed emitters from the experimental units and placed them at an adjacent site, where we removed caterpillars and visually estimated the leaf area consumed to verify that the intended levels of damage were achieved (estimates made by the same person, S. Díaz-Cruz) with prior training and calibration using images of damaged leaves and real levels of herbivory measured with the software Bioleaf (Machado *et al.*, 2016).

The day after removing emitters, we tested signalling effects on EFN induction (priming) for each receiver plant. For this, we placed one third-instar *A. argillacea* caterpillar for 48 h on each receiver and used serrated scissors to remove 25 % of tissue from two apical leaves per plant. We used a combination of natural and mechanical leaf damage because we did not have enough caterpillars to achieve the intended level of natural damage. We note, however, that mechanical damage has been shown to elicit a similar magnitude of EFN induction relative to natural herbivory in cotton (Wäckers and Wunderlin, 1999; Ramírez-Delgado *et al.*, 2025*a*). Mechanical damage was applied over two days consecutively (damaging one leaf per day), i.e. over the length of time that caterpillars were on plants. Importantly, the day before applying damage (after removing emitters), we shuffled the positions of receiver pairs, because EFN induction in damaged emitters could have increased ant attraction on neighbouring receiver plants, and this effect could be confounded with ant attraction to receivers via VOC-mediated inter-plant signalling. After this randomization, we spaced all receivers 1–1.5 m apart to minimize signalling between plants once damaged. We then conducted a survey of ant abundance on each plant at 72 h after damage onset on receiver plants. A total of 144 plants were examined, with each plant observed for 3 min. The survey was conducted between 06:00 and 09:00 h, which is when daytime ant activity is highest (S. Díaz-Cruz, personal observation).

We tested 71 plants for signalling effects on receiver resistance and ant-mediated effects on resistance by excluding ants on receiver plants from half of the experimental units the day after the second ant survey. This was done by applying Tanglefoot^®^ around the entire upper edge (2 cm-wide barrier) of the 2 L plastic bag containing each receiver plant and assigned to this treatment group, whereas for control (non-excluded) receiver plants we applied the barrier to half of the circumference to control for an effect of Tanglefoot application. Three weeks later, we visually estimated natural herbivory (percentage of leaf area removed, same methodology as above) on new leaves as a proxy of resistance to herbivory. The exclusion treatment proved to be effective, because virtually no ants were found on excluded receivers based on observations taking place during the following weeks (S. Díaz-Cruz, personal observation).

### Experiment 2: effects of signalling and relatedness on EFN induction and ant anti-herbivore responses

One month after measuring natural herbivory on receivers (Experiment 1), we conducted a second field experiment at the same site using the same design and methodology (unless stated otherwise) using a new set of plants (same age as in the previous experiment). Again, plant triplets were used, with one receiver with same mother as the emitter vs. another receiver with a different mother, and half of the emitters were induced with third or fourth instar *A. argillacea* caterpillars. Here, our goal was to obtain further insight into the plant responses behind ant recruitment and herbivore resistance patterns from Experiment 1. For this, we measured signalling effects on EFN induction and on ant recruitment (presumably, mediating ant-mediated resistance). Plants were the same age as for Experiment 1 (i.e. 2.5 months old). In this case, we included a total of 56 experimental units (*n* = 168 plants). At 48 h after damage onset, we collected VOCs from a subset of emitters (seven undamaged controls and eight damaged). As above, we removed emitters after 72 h of damage. After this VOC exposure period, receivers from 32 experimental units (*n* = 64 plants) were kept at the field site to test for ant responses to herbivore presence (probability of attack on sentinel caterpillars), whereas receivers belonging to the remaining 24 units (*n* = 48 plants) were transported to a nearby greenhouse at Unidad Multidisciplinaria de Docencia e Investigación of the Universidad Nacional Autónoma de México to test for effects on EFN induction. We moved this latter group of plants because EFN is difficult to measure *in situ* because of nectar loss attributable to evaporation and ant tending, and because sampling itself would affect nectar availability and, in turn, ant recruitment.

The day after moving receivers assigned to EFN measurements into the greenhouse, we induced both greenhouse and field plants on the same days (i.e. same amount of time after VOC exposure). At the greenhouse, plants were individually placed into anti-aphid mesh cages (60 cm in diameter, 80 cm high) covered on the outside with a plastic sheet (48.2 cm × 49.6 cm, Reynolds) to minimize effects of VOCs among cages (Briones-May *et al.*, 2023). Cages were spaced by 50 cm. Plants were induced by placing one *A. argillacea* caterpillar per plant on the second leaf (counting from the apical meristem downwards) for 48 h, and we additionally used serrated scissors to remove 25 % of the area of the fourth leaf over a 48 h period (see receiver induction procedures for Experiment 1). At 24 h after damage onset, we collected EFN from the first leaf for each plant. Then, 1 day after sampling EFN (72 h since damage onset), we collected the first leaf (counting from the apical meristem) to test effects on chemical defences (terpenoid aldehyde content), as a complementary means of (direct) defence that could inform resistance patterns in the field.

Receivers in the field were induced following the same procedures, and we conducted two bioassays to test ant anti-herbivore responses, one at 48 h and another at 72 h after the onset of receiver damage. For this, at each time point we fixed one fourth- or fifth-instar *A. argillacea* caterpillar on a leaf of each plant using an entomological pin. Caterpillars were pinned carefully through one lateral side and remained alive during the bioassays. After 1 h, we returned to the plants to record ant responses. Instances where the caterpillar was missing were not considered for statistical analysis (<5 % of cases). We estimated the probability of attack by ants separately for each bioassay (i.e. day) to test for attacks on a per 24 h basis. Bioassays were conducted between 06:00 and 08:00 h on both days.

### Trait measurements and quantification

We sampled the headspace of emitter plants *in situ* between 06:00 and 13:00 h, following Turlings *et al.* (1998). Briefly, whole plants, including damaged sections, were bagged within a nalophan bag, and VOCs were adsorbed on filters containing 25 mg of 80/100 mesh HayeSep-Q adsorbent (Sigma, Switzerland). One of the filter ends was inserted into the bag and the other end connected to a micro air sampler (Supelco PAS-500) at a flow rate of 200 mL min^−1^. For each sampling period, we also collected an air sample from empty bags, which served as an ambient control. After collecting volatiles for 2 h, traps were eluted with 150 μL dichloromethane, then spiked with 10 μL of internal standard solution (nonyl acetate, Sigma-Aldrich, Switzerland, 20 ng μL^−1^). Samples were sealed with polytetrafluoroethylene (PTFE) caps and Teflon tape, stored at −80 °C and sent to the laboratory of Fundamental and Applied Research in Chemical Ecology (FARCE) at the University of Neuchâtel (Switzerland) for gas chromatography–mass spectrometry analysis. Samples were analysed with a gas chromatograph (Agilent 7890B) coupled with a mass spectrometer detector (Agilent 5977B). A 1.5 μL aliquot of each sample was injected in pulsed splitless mode onto an Agilent HP-5MS column (30 m in length × 250 μm in diameter and 0.25 μm film thickness). After injection, the temperature was maintained at 40 °C for 3.5 min, increased to 100 °C at a rate of 8 °C min^−1^, and subsequently to 230 °C at a rate of 5 °C min^−1^ followed by a post run of 3 min at 250 °C. Helium was used as carrier gas and kept at a constant flow of 0.9 mL min^−1^. Compounds were subsequently identified by comparing their mass spectra with those from the NIST17 library and previous identifications for wild cotton VOCs. Peak areas were integrated using MZmine v.3.9.0 (Schmid *et al.*, 2023). Quantification was based on the relative response factors of a few commercial standards [(*Z*)-3-hexenal, (*E*)-2-hexenol, α-pinene, (*Z*)-3-hexenyl acetate, β-myrcene, (*E*)-β-ocimene, benzaldehyde, linalool, phenethyl acetate, methyl salicylate, indole, α-copaene, (*E*)-β-caryophyllene and (*E*)-β-farnesene from Sigma-Aldrich, Switzerland, and (*E*)-DMNT from Pherobank BV, the Netherlands] relative to nonyl acetate (Sigma-Aldrich, Switzerland), and measured in nanograms per hour.

For receiver EFN, we used 5 μL capillary tubes (Micropipettes Blaubrand^®^ intraMARK, Germany) to collect the EFN found on one fully or almost fully expanded undamaged leaf per plant. Nectar was collected between 06:00 and 07:00 h, and samples were taken to the laboratory to measure the amount (in microlitres) of EFN and its concentration, i.e. sugar content (expressed in degrees Brix, a measure of dissolved solids in a liquid) with a refractometer (Atago Master, Japan) (Briones-May *et al.*, 2023).

Finally, receiver leaf concentration of terpenoid aldehydes, namely gossypol and heliocides, was quantified at the FARCE laboratory and the Neuchâtel Platform of Analytical Chemistry (NPAC) at the University of Neuchâtel (Switzerland), in November 2023. Briefly, frozen leaves were ground under liquid nitrogen, and 50 mg of frozen leaf powder was extracted with 200 μL of a solution of acetonitrile, MilliQ water and formic acid (80:18.5:1.5). Samples were homogenized with three to five glass beads (1.25–1.65 mm in diameter) in a mixer mill for 3 min at 30 Hz (TissueLyser II, Qiagen, Germany) and ultrasonicated for 5 min. They were then centrifuged for 3 min at 8000*g*. The recovered supernatant was centrifuged a second time before being transferred to amber glass vials. Samples were analysed directly using ultra-performance liquid chromatography with diode array detection (UPLC-DAD, Ultimate 3000 Dionex, Thermo Fisher Scientific, MA, USA). The diode array detector was set at 288 ± 2 nm. A 10 μL sample was injected onto an ACQUITY UPLC^®^ BEH C18 column (2.1 mm × 100 mm, 1.7 μm; Waters, MA, USA). The flow rate was held constant at 0.45 mL min^−1^, and the temperature was kept at 40 °C. The mobile phase solvent A consisted of 0.05 % formic acid in MilliQ water (18 Ω) and the mobile phase solvent B of 0.05 % formic acid in acetonitrile (HiPerSolv, VWR Chemicals^®^, France). Solvent B increased from 45 to 90 % in 8 min, then to 100 % in 0.5 min, held at 100 % for 2.5 min, which was followed by re-equilibration at 45 % solvent B for 3.5 min. Gossypol and heliocides were identified by their respective retention times. Quantification was based on linear regression from six calibration points (5–250 μg mL^−1^) in gossypol equivalents. Concentrations were expressed in micrograms per gram of tissue on a fresh weight basis.

### Statistical analysis

Initially, we ran a linear mixed model (LMM) testing the effects of emitter herbivory (fixed) on emitter total VOC emissions. This model also included plant height as a covariate and the identity of the mother plant (treated as random). Total VOCs were log-transformed to achieve normality. In addition, we tested for changes in VOC composition (compound relative abundances) using a permutational analyses of variance (PERMANOVA) based on 999 permutations and using a Euclidean distance matrix based on individual compound proportions. Prior to running the PERMANOVA, we transformed VOC data using the centred log ratio (Hervé *et al.*, 2018). The model included herbivory as a main effect, plant height as a covariate, and mother plant. To visualize these results, we performed a principal coordinate analysis based on Euclidean pairwise dissimilarities (Moreira *et al.*, 2021). Finally, for both the LMM and the PERMANOVA, we included experiment as a fixed effect because VOC data used in these analyses were collected during both experiments (see above). In addition, to explore the association between VOC emission similarity and plant genetic relatedness, we performed Mantel tests comparing pairwise dissimilarity matrices of VOC profiles among families based on data from emitter plants with a matrix of genetic distances among families (see Supplementary Data Table S1). This analysis was done separately for VOC data from damaged and control emitters (induced and constitutive emissions, respectively).

For receiver responses, we tested for an effect of emitter herbivory treatment (fixed), emitter–receiver relatedness (same vs. different mother, fixed) and their interaction (fixed) on the survey of ant abundance (number of ants), probability of ant attack on sentinel caterpillars (hereafter referred to as probability of attack), EFN volume and concentration, in addition to the concentration of gossypol and heliocides using generalized linear mixed models (GLMMs). Models for nectar, resistance and secondary metabolites used a Gamma distribution (log link), the GLMM for ant abundance used a negative binomial distribution (log link), and attack GLMM used a binomial distribution (logit link). All models included the experimental unit (i.e. plant triplet) and the identity of the mother plant of emitters and receivers as random effects, and we also included the percentage of experimentally imposed leaf area removed on receivers as a covariate to account for dose dependence in defence (VOCs) induction. In the case of the percentage of natural herbivory (to assess effects on plant resistance; Experiment 1), we fitted a zero-inflated beta GLMM because 40 % of plants showed no herbivory. This model included three components: (1) a conditional component modelling herbivory proportion on attacked plants using a beta distribution with a logit link; (2) a zero-inflated component modelling the probability of absence of herbivory using a binomial distribution with a logit link; and (3) a dispersal component allowing for differential variability among treatments. As above, main (fixed) effects included were emitter herbivory treatment, emitter–receiver relatedness and ant exclusion treatment, in addition to their two-way interactions and the three-way interaction. We included as random effects the experimental unit and the identity of the mother plants of emitter and receiver plants.

All analyses were run in R v.4.4.1 (R Core Team, 2024). The LMM and GLMMs were implemented with the *lmer* and *glmmTMB* functions of the *lme4* and *glmmTMB* packages, respectively (Bates *et al.*, 2015; Brooks *et al.*, 2017). The *DHARMa* package was used to evaluate model fit (Hartig, 2014). Effects of the explanatory variables were tested using the *lmerTest* package for LMMs (Kuznetsova *et al.*, 2017) and the *car* package for GLMMs (Fox and Weisberg, 2019). Model least-squares means and standard errors were obtained with the *lsmeans* function of the package *emmeans* (Lenth, 2024). For multivariate analyses of VOC profiles, dissimilarity matrices were calculated using the *vegdist* function, and Mantel tests were performed with the *mantel* function. PERMANOVA and ordination analyses were conducted using the *adonis2* and *capscale* functions, respectively. All multivariate functions are part of the *vegan* package (Oksanen *et al.*, 2025).

## RESULTS

### Plant genetic relatedness

The mean inter-sibling genetic distance varied three-fold between family lineages (Supplementary Data Table S1), which is similar to the range of inter-sibling distances within families (i.e. two- to three-fold, except for one family, in which inter-sibling distances varied nearly 11-fold; Supplementary Data Table S1). As expected, genetic distance between plants from different families was generally greater than inter-sibling distances, with mean inter-family distance varying ≤2-fold among comparisons (Supplementary Data Table S1 and Fig. S1); however, inter-individual (non-familial) distances were slightly more variable (Supplementary Data Fig. S1), ranging up to 4-fold.

### Effects of herbivory on cotton volatile emissions

We identified a total of 39 compounds in the headspace of emitter plants (Supplementary Data Table S2). There was a significant effect of the emitter herbivory treatment (Table 1); damaged plants emitted on average 3.3-fold greater amounts of total VOCs (1232.99 ± 192.10 ng h^−1^) compared with control plants (374.24 ± 66.56 ng h^−1^). When testing differences in individual compounds, (*E*)-β-ocimene, (*E*)-DMNT, β-farnese, indole and (*E*)-β-caryophyllene were substantially more emitted from damaged plants. Consistently, these five compounds have previously been shown to be highly inducible in cotton (Supplementary Data Table S3). In addition, there was ≤9-fold variation among mother plants in total VOC emissions for undamaged plants (i.e. constitutive state) and ≤3-fold variation among mother plants for damaged plants (i.e. induced state) (Supplementary Data Fig. S2). In addition, the PERMANOVA indicated a significant effect of herbivory on VOC composition (Table 1). The first two axes of the principal coordinate analysis explained 70.4 % of the variation (62.43 % and 7.97 %, respectively; Supplementary Data Figs S3 and S4). Finally, there was no significant correlation between the genetic relatedness among mother plants and their VOC compositional dissimilarity, and this was consistent using VOC data from both control (Mantel *r* = −0.023, *P* = 0.51) and induced (Mantel *r* = −0.14, *P* = 0.63) emitters (Supplementary Data Fig. S5).

**Table 1.** Results from an LMM and a PERMANOVA testing the effect of emitter herbivory treatment (fixed, damaged vs. undamaged) using the specialist caterpillar *Alabama argillacea*, both on total VOCs emitted (in nanograms per hour) and on VOC composition (based on compound relative abundances) released by wild cotton (*Gossypium hirsutum*) emitter plants. The model for total VOCs included plant height as a covariate, and both models also included experiment (fixed; see the Materials and Methods) and mother plant (random in the LMM, statistics not shown). Shown are test statistics, degrees of freedom, and significant values for each effect. Significant effects (*P* < 0.05) are show in bold.

Effect	Total VOC emissions			VOC composition	
	*F*	d.f.	*P*-value	Pseudo-*F*	*P*-value
Emitter herbivory	33.75	1, 49	**<0.0001**	6.408	**0.001**
Experiment	1.82	1, 48	0.10	4.398	**0.001**
Emitter height	2.4	1, 52	0.12	–	–

#### Experiment 1: signalling and plant relatedness effects on ant abundance and herbivory resistance

**Fig. 2.**
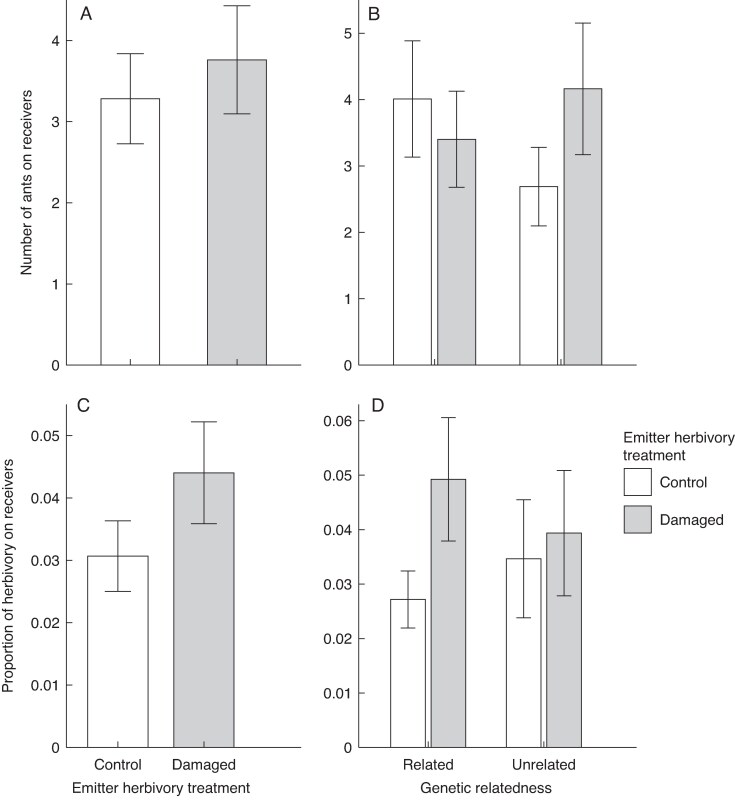
Main effect of wild cotton (*Gossypium hirsutum*) emitter herbivory treatment (damage by the specialist *Alabama argillacea* vs. undamaged) and of this treatment under each level of emitter–receiver relatedness on the abundance of ants (A and B) and on the percentage of leaf area removed owing to natural herbivory on newly produced leaves (post-damage) by receiver plants (C and D). Shown are back-transformed least-squares means and standard errors from a negative binomial model.

A total of 1462 ants belonging to 10 species were recorded on experimental cotton plants. The most abundant species were *Brachymyrmex australis* (58.48 %), *Forelius pruinosus* (17.03 %), and *Pheidole* sp. (11.42 %) (Supplementary Data Table S4). We found no significant effects of emitter herbivory treatment, emitter–receiver relatedness, or their interaction on the number of ants recorded on receivers (Table 2; Fig. 2A, B).

**Table 2.** Results from a generalized (negative binomial) linear mixed model testing the effects of emitter herbivory (fixed, undamaged vs. damaged by the specialist caterpillar *Alabama argillacea*), emitter–receiver relatedness (fixed, same vs. different mother plant). and their interaction on the number of ants on wild cotton (*Gossypium hirsutum*) receiver plants. The model also included as a covariate the percentage of leaf area consumed owing to experimentally imposed herbivory on receivers. The identities of the emitter and receiver mother plant, in addition to the plant triplet, were included as random effects (statistics not shown). Shown are test statistics (χ^2^), degrees of freedom, and significance values for each effect.

Effect	Ant abundance		
	χ^2^	d.f.	*P*-value
Emitter herbivory (*H*)	0.29	1	0.58
Emitter–receiver relatedness (*R*)	0.26	1	0.61
*H* × *R*	2.30	1	0.13
Receiver herbivory	0.48	1	0.49

Cumulative natural herbivory on new leaves produced 3 weeks after exposing receivers was low (grand least-squares mean ± s.e.: 2.20 ± 0.45 %). The zero-inflated model indicated no significant effect of the emitter herbivory treatment, plant relatedness or ant exclusion on the amount of subsequent natural herbivory (Table 3; Fig. 2C). The covariate for herbivory experimentally imposed on receivers was also not significant. Furthermore, there were no significant two- or three-way interactions between any of these factors (Table 3; Fig. 2D).

**Table 3.** Results from a generalized (zero-inflated) linear mixed model testing the effects of emitter herbivory (fixed, undamaged vs. damaged by the specialist caterpillar *Alabama argillacea*), emitter–receiver relatedness (fixed, same vs. different mother plant) and their interaction on the percentage of leaf area lost to natural herbivory for new leaves produced three weeks after treatment application (i.e. less damage indicates greater resistance) by wild cotton (*Gossypium hirsutum*) receiver plants. The model also included as a covariate the percentage of herbivory experimentally imposed to receivers (to test for priming effects on induction on these plants), in addition to the effect of receiver ant exclusion (to tease apart the effect of ant-mediated indirect defence on plant resistance; see the Materials and Methods) and their interactions with the above-mentioned main effects. Shown are test statistics (χ^2^ values), degrees of freedom, and significance values for each effect.

Effect	Natural herbivory on receiver new leaves		
	χ^2^	d.f.	*P*-value
Emitter herbivory (*H*)	3.17	1	0.075
Emitter–receiver relatedness (*R*)	0.05	1	0.82
Receiver ant exclusion (*E*)	0.93	1	0.33
Receiver (imposed) herbivory	0.11	1	0.74
*H* × *R*	0.78	1	0.37
*H* × *E*	0.34	1	0.56
*R* × *E*	0.84	1	0.36
*H* × *R* × *E*	0.35	1	0.55

#### Experiment 2: signalling and plant relatedness effects on EFN and caterpillar attacks by ants

**Fig. 3.**
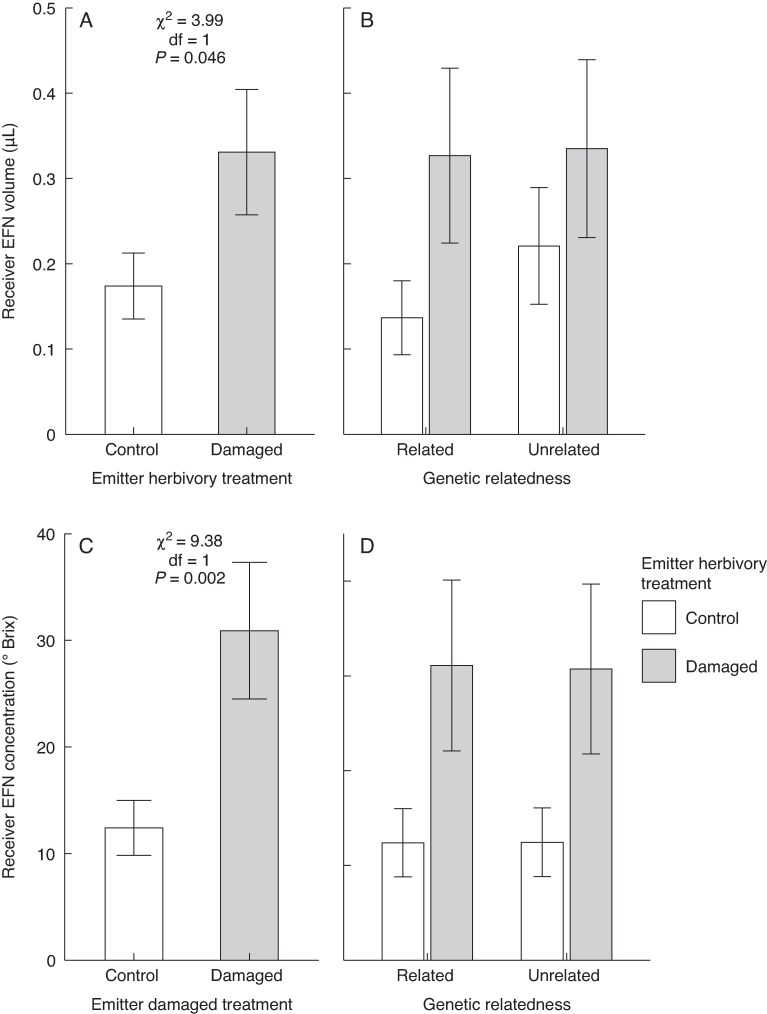
Main effect of wild cotton (*Gossypium hirsutum*) emitter herbivory treatment (damaged by the specialist *Alabama argillacea* vs. undamaged) and of this treatment under each level of emitter–receiver relatedness on extrafloral volume in microlitres (A and B) and concentration in dgrees Brix (C and D) collected from induced receiver plants. Shown are model least-squares means and standard errors. Statistics (χ^2^, d.f. and *P*-values) are shown only for significant model terms (*P* < 0.05).

We found a significant effect of emitter herbivory treatment on receiver EFN volume (Table 4), whereby receiver plants exposed to damaged emitters produced, on average, nearly a two-fold greater amount of nectar in response to damage (0.330 ± 0.07 μL) compared with those exposed to control emitters (0.174 ± 0.04 μL) (Fig. 3A). Likewise, we also found a significant effect of emitter herbivory on receiver EFN concentration (Table 4), with receivers exposed to damaged emitters producing, on average, EFN with a 2.5-fold greater mean concentration of sugar after damage (30.9 ± 6.41 ° Brix) compared with those exposed to control emitters (12.4 ± 2.57 ° Brix) (Fig. 3B). However, there was no significant main effect of emitter–receiver relatedness (Table 4) and no significant emitter herbivory–relatedness interaction for either EFN volume or sugar concentration (Table 4; Fig. 3C, D). Lastly, there were no significant effects of emitter herbivory, emitter–receiver relatedness, or an interaction for either gossypol or heliocides (Supplementary Data Table S5).

**Table 4.** Results from generalized linear mixed models testing for effects of emitter herbivory treatment (fixed, undamaged vs. damaged by the specialist caterpillar *Alabama argillacea*), emitter–receiver relatedness (fixed, same vs. different mother plant) and their interaction on the amount (volume, in microlitres) and concentration (in degress Brix) of extrafloral nectar produced by wild cotton (*Gossypium hirsutum*) receiver plants (gamma models with log link function), in addition to the attack rate by ants (24 h probability of attack recorded on twodays consecutively) on *Alabama argillacea* caterpillars placed on receivers (binomial model with logit link function). Both models also included the percentage of leaf damage experimentally imposed on receivers as a covariate, along with the effect of ant exclusion and its interactions with the main effects. Shown are the test statistics (χ^2^), degrees of freedom and significance values for each effect. Significant (*P* < 0.05) effects are in bold.

	Extrafloral nectar						Ant attack rate					
	Volume			Concentration			Survey 1			Survey 2		
Effect	χ^2^	d.f.	*P*-value	χ^2^	d.f.	*P*-value	χ^2^	d.f.	*P*-value	χ^2^	d.f.	*P*-value
Emitter herbivory (*H*)	3.99	1	**0.046**	9.38	1	**0.002**	0.83	1	0.36	3.84	1	**0.049**
Relatedness (*R*)	0.65	1	0.42	0.0002	1	0.99	0.02	1	0.89	1.35	1	0.22
*H* × *R*	0.69	1	0.40	0.0007	1	0.98	0.02	1	0.88	0.02	1	0.87
Receiver herbivory	0.52	1	0.69	2.24	1	0.13	0.47	1	0.49	6.38	1	**0.011**

**Fig. 4.**
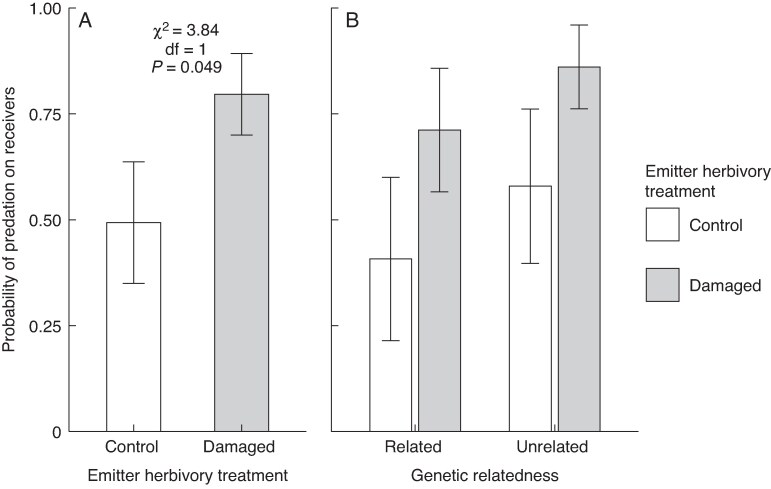
Main (overall) effect of wild cotton (*Gossypium hirsutum*) emitter herbivory treatment (undamaged vs. damaged by the specialist caterpillar *Alabama argillacea*) (A) and of this treatment under each level of emitter–receiver relatedness (B) on the probability of attack on sentinel caterpillars by ants on induced receiver plants (data for survey 2; see Results). Shown are back-transformed least-squares means and standard errors from a binomial model. Statistics (χ², d.f. and *P*-values) are shown only for significant model terms (*P* < 0.05).

We found no significant effect of emitter herbivory treatment (control emitters, 0.80 ± 0.09; damaged, 0.70 ± 0.10), emitter–receiver relatedness or their interaction on the probability of attack during the first survey (Table 4). However, during the second survey we found a significant effect of the emitter herbivory treatment (Table 4), whereby caterpillars placed on receivers exposed to damaged emitters had a 1.5-fold greater probability of attack (0.77 ± 0.07) compared with caterpillars found on receivers exposed to undamaged emitters (0.50 ± 0.11) (Fig. 4A). The most common species attacking caterpillars were *F. pruinosus*, *B. australis* and *Pheidole yucatana*. Again, however, there was no significant effect of emitter–receiver relatedness (Table 4) and no significant interaction between emitter herbivory and relatedness on the probability of attack (Table 4; Fig. 4B).

## DISCUSSION

### Induction of wild cotton volatiles

Herbivory by the specialist *A. argillacea* significantly induced wild cotton volatile emissions, causing both quantitative (total VOCs) and qualitative (i.e. VOC composition) changes. We also found evidence that several ecologically important compounds were significantly induced, including (*E*)-β-ocimene, (*E*)-4,8-dimethyl-13,7-nonatriene ((*E*)-DMNT), (*E*)-β-farnesene, indole, and (*E*)-β-caryophyllene. This agrees with previous work on cotton, which mostly used cultivated genotypes (e.g. Loughrin *et al.*, 1994; Grandi *et al.*, 2024), but also recent work with wild cotton (e.g. Mamin *et al.*, 2025). Several of these compounds have been implicated in parasitoid and predator attraction in other plants, such as (*E*)-β-farnesene in *Arabidopsis thaliana* (Kutty and Mishra, 2023), (*E*)-DMNT in *Zea mays* (Turlings *et al.*, 1995), (*E*)-β-caryophyllene in *Nicotiana attenuata* (Kessler and Baldwin, 2001), and both (*E*)-β-farnesene and (*E*)-β-caryophyllene in *Acacia cochliacantha* (Hernández-Zepeda *et al.*, 2018). Likewise, some compounds are likely to mediate inter-plant signalling; for example, (*E*)-β-ocimene, (*E*)-β-caryophyllene and indole play crucial roles in activating defence priming, enhancing the resistance of nearby plants to herbivory for plants such as tobacco and maize (Arimura *et al.*, 2012; Erb *et al.*, 2015; Taggar *et al.*, 2024). *De novo* synthetized induced volatiles also appear to play a role in signalling interactions between cultivated cotton plants (Grandi *et al.*, 2024), and field trials with mechanically damaged cotton suggest that their volatiles can induce significant resistance to pest insects in neighbouring plants (Renou *et al.*, 2011; Llandres *et al.*, 2018). In the case of wild cotton, further work is needed to test the importance of inducible volatiles in within- and between-plant signalling.

Importantly, we found substantial variation in total VOC emissions among the mother plants. There are studies reporting on plant genetic variation in volatile emissions, particularly work with maize (Degen *et al.*, 2004), *Solidago altissima* (Kalske *et al.*, 2019), and *Solanum tuberosum* (Agho *et al.*, 2023). Nonetheless, fewer studies have been conducted in wild plants, and rarely has such variation been linked to plant-to-plant signalling and associated interactions. One exception is a study showing that genotypic variation in VOC emissions in barley (*Hordeum vulgare*) cultivar mixtures affected aphid attraction (Dahlin *et al.*, 2018). In addition, recent work has shown that the ecological context shapes the magnitude of plant genotypic variation in volatile emissions, e.g. plants might converge towards similar volatile emissions in environments with high herbivory (*S. altissima*: Kalske *et al.*, 2019; *A. tridentata*: Karban *et al.*, 2024). This could reflect an adaptive response whereby generalized signalling channels are selected to achieve more widespread inter-plant defence induction (i.e. the mutual benefit hypothesis; Kalske *et al.*, 2019). For wild cotton, previous work by our group has shown a high degree of population-level variation in leaf volatile content (e.g. relative amounts of monoterpenes and sesquiterpenes), and that such variation is heritable (Clancy *et al.*, 2023). There is also evidence of chemotypic variation among and within populations (Clancy *et al.*, 2023; Mamin *et al.*, 2025). Further research is needed to tease apart sources of genetic variation in volatile emissions (e.g. chemotypic vs. other sources of genetic variation) within and among populations to gain a better understanding of its drivers and ecological relevance.

### Signalling effects on extrafloral nectar induction

Receiver plants exposed to damaged emitters during Experiment 2 exhibited a more concentrated and larger volume of EFN after being damaged in comparison to plants exposed to undamaged emitters. This signalling effect agrees with previous work on this species (Briones-May *et al.*, 2023), corroborating the importance of herbivory-induced VOCs in priming EFN induction. Similar inter-plant signalling effects on EFN have been reported in at least one other species, i.e. lima bean *P. lunatus* (Heil and Silva-Bueno, 2007), but overall remain poorly understood for EFN induction in addition to other plant indirect defence traits (Heil and Karban, 2010; Turlings and Erb, 2018). Contrary to previous work showing that VOC-mediated signalling effects are stronger when emitter and receiver plants are more closely related (e.g. Karban *et al.*, 2013, 2014; Moreira *et al.*, 2016; Grof-Tisza *et al.*, 2021, 2022) and consistent with our findings, other studies have found no evidence of genetic relatedness affecting VOC-mediated signalling. For example, despite substantial qualitative and quantitative genotypic variation in VOC emissions, relatedness did not influence signalling effects related to *S. exigua* resistance in potato (*S. tuberosum*; Martín-Cacheda *et al.*, 2023). This suggests, on the one hand, that there is some degree of plant inter-specific variation in signalling effects as a function of specific ecological and evolutionary dynamics prevalent in each system (e.g. in herbivore pressure; see also Pearse *et al.*, 2012). On the other hand, it also suggests that the response in neighbouring plants to certain compounds or groups of compounds is relatively stable (at least within certain limits) despite observed quantitative and qualitative variation in induced VOCs.

The fact that herbivory is highly variable among wild cotton plants or populations (and often low) would predict more closed signalling channels and the emergence of plant relatedness effects (Grof-Tisza *et al.*, 2021). This is not what we found. We observed no correlation between plant relatedness and VOC dissimilarity, suggesting that closely related plants do not necessarily have more effective or exclusive communication channels. Instead, they appear to share information openly with unrelated individuals, consistent with the mutual benefits hypothesis (Kalske *et al.*, 2019). Alternatively, limiting or encrypting signals to prevent communication with other plants might be too costly for emitters, particularly if there are no significant drawbacks to sharing these signals, making such restrictive strategies unlikely to evolve (Scott *et al.*, 2025). That said, it is worth pointing out that for EFN concentration there was an 11- vs. three-fold induction when emitter–receiver pairs were of the same vs. different mother plant, respectively (Fig. 3D). Although this difference in magnitude of effect was not statistically significant, it calls for more in-depth efforts using a larger number of wild cotton mother plants from more distant populations to increase variability and more robustly test plant relatedness or, more broadly, different sources of genetically based (including chemotype-related variation; see above) effects on inter-plant signalling.

### VOC signalling effects on ant–cotton interactions

Inconsistent with EFN results, we found no effect of emitter herbivory treatment (i.e. signalling) on ant abundance on receiver plants during Experiment 1. This suggests that signalling effects on EFN induction were either not strong enough to influence ant attraction (but did affect the probability of attack; see discussion below) and/or that other intervening factors *in situ* weakened such an effect on ant recruitment. In contrast, Kost and Heil (2006) detected increased ant and wasp attendance following VOC exposure in *P. lunatus*, indicating that neighbour volatile-triggered effects on receiver EFN can attract defensive mutualists in certain conditions. Work in the coastal wild cotton–ant system in Yucatan has shown that ant foraging patterns are strongly correlated with nectar secretion (Ramírez-Delgado *et al.*, 2025*b*). Accordingly, we surveyed ant abundances soon after ending receiver VOC exposure and induction, which is when effects on ant attraction and responses to EFN were expected to be strongest. Nonetheless, the short duration of observations and having conducted a single survey could have limited our ability to detect brief time windows of high ant activity (during that day or the day before/after), particularly given high fluctuations in ant abundances occurring at fine temporal scales presumably common to ant–plant interactions on wild cotton and reported for other plant species (e.g. Liu *et al.*, 2020; Guimarães, *et al.*, 2023). That said, it is also possible that induced VOCs in receivers directly affected ant attraction (Mobarak *et al.*, 2025), a possibility that merits further attention given emerging evidence for these VOC-mediated dynamics in other ant–plant systems. Accordingly, one challenge in future work is to tease a part signalling effects on EFN from those attributable to VOCs and the relative contributions in different ecological contexts (Schettino *et al.*, 2017; reviewed by Nelson *et al.*, 2019; see discussion by Moreira *et al.*, 2024). Lastly, our study did not look at ant nocturnal activity (see Guimarães, *et al.*, 2023). Given that induced VOCs (peaks and cycles in production or emissions) are mainly associated with daytime and fall during the night (Loughrin *et al.*, 1994), it is unlikely that they would mediate cotton–ant interactions during the night. However, EFN night-time production has been reported in other species (e.g. cacti; Holland *et al.*, 2010; reviewed by Gaffal, 2012) and represents a pending aspect to study for wild cotton.

In contrast, we found that the emitter herbivory treatment caused a significant 1.5-fold greater probability of caterpillar attack by ants (mostly *F. pruinosus*, *B. australis* and *P. yucatana*) on induced receiver plants exposed to damaged emitters relative to those exposed to undamaged emitters (Experiment 2, bioassay 2). This result is consistent with the signalling effect on EFN, strongly suggesting that signalling effects on attack on caterpillars by ants were mediated by changes in EFN. Previous work reporting on VOC signalling effects on EFN and plant resistance (again, *P. lunatus*: Heil and Silva-Bueno, 2007) did not measure predator responses or test for plant relatedness effects (see below). Doing so is key not only to link indirect defence trait and resistance outcomes but also to gain insight into how VOC signalling affects top-down trophic control. Although we cannot reject the possibility that effects on ant abundance went undetected (see above) or that, for example, ant composition changed between experiments (abundance and predation were measured in Experiment 1 and 2, respectively), including the presence of more aggressive ant species, such as *Pheidole* sp., during the second bioassay (e.g. see Liu *et al.*, 2020), results thus far suggest that increased probability of attack might not attributable to higher ant abundance (i.e. a density-mediated effect) but rather to changes in ant behaviour (Abdala-Roberts and Mooney, 2015). This interpretation aligns with the ‘nutritional deficit hypothesis’ proposed by Ness *et al.* (2009), which posits that access to carbohydrate-rich resources, such as EFN, can increase ant aggressiveness towards herbivores by creating a nutritional imbalance that heightens their demand for nitrogen-rich protein.

Interestingly, during bioassay 1 we observed no differences in the probability of attack between receivers exposed to damaged vs. control emitters, but those rates were relatively high in both treatments (see Results). We speculate that differences in nectar might have been smaller on the first day after damage, because plants typically produce larger amounts of EFN immediately after damage, particularly when watering is more recent (Wäckers *et al.*, 2001). By the second day (survey 2), although absolute EFN production might have declined, the difference in EFN between receivers exposed to damaged versus undamaged emitters might have become more pronounced, explaining the significant difference in caterpillar probability of attack observed only in survey 2. This temporal dynamic in EFN production and the magnitude of treatment differences could also account for the contrasting results between surveys. Non-exclusively, there might have been a time lag in ant recruitment since receiver damage onset, which resulted in a weaker discrimination between receiver plants exposed to control vs. damaged emitters during bioassay 1.

### Signalling effects on resistance to herbivory

We found no evidence for signalling effects on resistance to natural herbivory over a three week time span in Experiment 1, nor was there an effect of ant exclusion during this period. This is in agreement with previous work showing weak or no evidence of ant-mediated protection against insect herbivores and pathogens in wild cotton from Yucatan (Abdala-Roberts *et al.*, 2019*b*; Reyes-Hernández *et al.*, 2022), albeit with differences in ant abundance and composition across studies. For example, Reyes-Hernández *et al.* (2022) reported a high abundance of *Crematogaster* sp. at a tropical forest site, whereas in the present study the ant community on cotton plants was dominated by *B. australis* and *F. pruinosus*, common species at some coastal shrubland sites (Ramírez-Delgado *et al.*, 2025*b*). Low herbivory levels (<2 % of leaf area consumed, on average) expectedly limited the test of signalling effects on ant-provided protection and the effect of ant exclusion (or direct chemical defences, for that matter). Herbivory on wild cotton can be considerably low and sporadic at some sites, as was the case during our study, suggesting that VOC signalling effects on ant–plant interactions are highly variable and context dependent, a condition that is intrinsic to ant–plant interactions in many systems (Chamberlain *et al.*, 2014), including other *Gossypium* species (e.g. *Gossypium thurberi*; Rudgers *et al.*, 2004; Chinarelli *et al.*, 2022). That said, recent work by our group suggests that ant-mediated protection can be important in some contexts, e.g. when herbivory is high and dispersed (rather than concentrated) across leaves, leading to local induction of more nectaries (Ramírez-Delgado *et al.*, 2025*b*). We currently ignore how strong or long-lived these effects are to significantly affect indirect resistance and plant fitness. Importantly, a key factor to test in addressing context-dependencies in EFN induction and ant–plant interactions is soil salinity, which has been shown to weaken signalling effects on EFN induction (Briones-May *et al.*, 2023) and is expected to vary considerably both within and among wild cotton populations. In contrast, given that defensive terpenoid aldehydes were not induced by herbivory in neighbours, it would not be expected that direct defences in receiver plants would play a role in reducing herbivory. Previous work in cultivated cotton has shown that exposure to induced VOCs rapidly increases jasmonates in leaves, whereas induction of terpenoid aldehydes is minimal (Grandi *et al.*, 2024). In damaged plants, gossypol takes from 1–2 weeks to accumulate in new leaves (Bezemer *et al.*, 2004; Eisenring *et al.*, 2017), hence the sampling timing used for Experiment 2 might not have been optimal to observe induction in these chemical defences. Work is currently underway to investigate the effects of abiotic forcing attributable to soil salinity on EFN-mediated interactions, including local adaptation of wild cotton to soil conditions across contrasting edaphic microhabitats to get at evolutionary implications.

### Conclusion

We herein provide strong evidence that inter-plant signalling influences top-down effects on herbivores by ants via EFN. The short duration of our study combined with low and highly variable herbivory rates points to context dependence in downstream consequences for plant indirect resistance, calling for work assessing spatial and temporal variation in these dynamics in ways that help to identify the biotic and abiotic conditions in which these interactions are likely to matter most for wild cotton. In particular, determining the conditions in which VOC signalling influences ant deterrence or predation on herbivores and whether this feeds back to benefit wild cotton resistance and reproduction are key challenges for future work. In doing so, expanding on ant foraging behaviour could help to explain results in facultative ant–plant mutualisms, such as that with wild cotton, where ants do not inhabit the plant and their abundances fluctuate strongly and can be low. All in all, knowledge gained from these endeavours could also have spill-over implications for understanding indirect defence in cultivated cotton, given the recent work showing EFN-mediated indirect defence by ants in cultivated varieties of upland cotton (Llandres *et al.*, 2019). To do so, studies must consider specific cultivation contexts, where arthropod communities vary considerably from those on wild cotton (e.g. aphids and exotic ants; Kaplan and Eubanks, 2005), including players that are much more abundant or exclusive to cotton agroecosystems.

## Supplementary Material

mcaf315_Supplementary_Data

## Data Availability

The data from all analyses is available in Zenodo at https://doi.org/10.5281/zenodo.1802206. Sequences were deposited at NCBI (PRJNA1280973).

## SUPPLEMENTARY DATA

Supplementary data are available at *Annals of Botany* online and consist of the following. Section S1: methodological procedure for sample processing and genomic analyses to obtain genetic relatedness data from the mother plants and siblings used in the experiments. Table S1: comparison of pairwise genetic distances within and among families. Table S2: descriptive statistics of volatile organic compounds emitted by wild cotton damaged and undamaged. Table S3: results from models testing for the effects of leaf damage treatment on the emission of different volatile organic compounds from emitter cotton plants. Table S4: list of ant species recruiting to wild cotton. Table S5: results of linear mixed-effects model testing for effects of emitter herbivory and relatedness on defensive metabolites. Figure S1: heat map of genetic distance between and among families. Figure S2: total VOC emissions across wild cotton mother plants for control and damaged plants. Figure S3: effect of the emitter herbivory treatment on compositional variation in VOC emissions based on a principal coordinates analysis using compound relative abundances for control and damaged wild cotton plants. Figure S4: variation in VOC compositions among siblings of each mother plant based on a principal coordinates analysis using compound relative abundances. Figure S5: correlation (Mantel tests) between genetic distance and VOC compositional dissimilarity across mother plants.
