# Supplementary material for: Tri-trophic consequences of plant-to-plant volatile signalling and its contingency on plant relatedness in wild cotton
Source: Ann Bot. 2025 Dec 8;137(4):985–1000. doi: 10.1093/aob/mcaf315 (PMC13095879; doi:10.1093/aob/mcaf315)
Supplement: mcaf315_Supplementary_Data [file mcaf315_supplementary_data.docx]

**SUPPLEMENTARY DATA**

Tri-trophic consequences of plant-to-plant volatile signaling and its contingency on plant relatedness in wild cotton

Sandra Díaz-Cruz, Ted. C. J. Turlings, Uriel Solís-Rodríguez, Jonathan Interian-Aguiñaga, Víctor Hugo Ramírez-Delgado, Mary V. Clancy, Marine Mamin, Jonathan F. Wendel, Corrinne E. Grover, Mark A. Arick II, Chuan-Yu Hsu, Olga Pechanova, Adam Thrash, Daniel G. Peterson, Carlos Bustos-Segura, Luis Abdala-Roberts

**Section S1.** Sample processing and genomic analyses to obtain genetic relatedness data from the mother plants and siblings used in the experiments. Briefly, the genomic DNA extraction and QC validation were conducted using 30 to 50 mg of dry leaf tissues from each cotton sample based on the previously published procedure (Ning et al., 2024). Approximately 350 ng of genomic DNA per sample was used for the ddRAD-Seq library construction followed by a protocol described by Magbanua et al., (2023) using *Hind*III (New England Biolabs, Ipswich, MA, USA) as a rare cutter restriction enzyme and *Bfa* I (New England Biolabs, Ipswich, MA, USA) as a frequent cutter restriction enzyme. The quality and quantity of each barcoded library was assessed using Agilent Bioanalyzer 2100 system (Agilent Technologies, Santa Clara, CA) and Qubit fluorometer (Thermo Fisher Scientific, Waltham, MA), respectively. A total of 91 barcoded libraries were equimolarly multiplexed into a single pool. The genetic relationships underlying the sibling cohorts and between families were measured using a ddRAD-seq survey of ten representatives for each family, including the maternal parent. An average of 129.2 Mb (range: 100.5 - 131.0 Mb) of sequence was recovered from each sample, representing approximately 5.6% of the genome. The obtained pooled library sample was sent to Novogene (Sacramento, CA, USA) for sequencing on the NovaSeq X Plus (Illumina, San Diego, CA). The overlapping pairs of raw reads for each sample were merged using vsearch (v2.29.0; Rognes et al., 2016). The merged reads were trimmed and filtered using cutadapt (v4.9; Martin 2011), removing any read without both restriction enzymes. The filtered data were aligned to the TX2094 reference genome using bwa (v0.7.18; Li 2013) and variants were called using the Stacks pipeline (v2.68; Rochette et al., 2019). The resulting variants were analyzed in R (v4.2.2; R Core Team 2024) using the packages SNPRelate (v1.32.2; Zheng et al., 2012), vcfR (v1.15.0, Knaus and Grünwald 2017), ape (v5.7-1, Paradis and Schliep 2019) and tidyverse (v2.0.0; Wickham et al., 2019) to filter variants, calculate genetic distance and relatedness, compute basic statistics, and generate plots. All code used in the analysis can be found at https://github.com/IGBB/mexico-cotton-ddrad/.

**Table S1.** Comparison of pairwise genetic distances within (above line) and among (below line) families. Statistics (mean, median, and range) are computed for each family both including (Mom) and excluding (Kid) the maternal plant. Methods are described in Appendix S1: Section 1.

| **Comparison** | **Mean (mom)** | **Mean (kid)** | **Median (Mom)** | **Median (Kid)** | **range (Mom)** | **range (Kid)** |
| --- | --- | --- | --- | --- | --- | --- |
| CH1S54 - CH1S54 | 0.0108 | 0.0113 | 0.0108 | 0.0111 | 0.0052 - 0.0164 | 0.0062 - 0.0164 |
| CH2S52 - CH2S52 | 0.0111 | 0.0111 | 0.0081 | 0.0081 | 0.0056 - 0.0243 | 0.0056 - 0.0243 |
| CONM10 -CONM10 | 0.0119 | 0.0126 | 0.0095 | 0.0168 | 0.0022 - 0.0238 | 0.0022 - 0.0238 |
| CONS51 - CONS51 | 0.014 | 0.0129 | 0.0139 | 0.013 | 0.0074 - 0.0196 | 0.0074 - 0.0165 |
| CONS52 - CONS52 | 0.0118 | 0.0124 | 0.0112 | 0.0142 | 0.0053 - 0.0179 | 0.0068 - 0.0179 |
| LAGS53 - LAGS53 | 0.0093 | 0.0097 | 0.0082 | 0.0083 | 0.0046 - 0.0152 | 0.0066 - 0.0152 |
| LAGS58 - LAGS58 | 0.0128 | 0.0134 | 0.0121 | 0.0127 | 0.0063 - 0.0215 | 0.0074 - 0.0215 |
| VGS51 - VGS51 | 0.0046 | 0.0046 | 0.0046 | 0.0046 | 0.0033 - 0.0059 | 0.0033 - 0.0059 |
| VGS53 - VGS53 | 0.0105 | 0.0105 | 0.0097 | 0.0097 | 0.0062 - 0.0163 | 0.0062 - 0.0163 |
| CH1S54 - CH2S52 | 0.0282 | 0.0279 | 0.0281 | 0.0279 | 0.0255 - 0.0318 | 0.0255 - 0.0302 |
| CH1S54 - CONM10 | 0.027 | 0.0267 | 0.0265 | 0.0262 | 0.0236 - 0.0320 | 0.0236 - 0.0320 |
| CH1S54 - LAGS53 | 0.0249 | 0.0245 | 0.0247 | 0.0244 | 0.0218 - 0.0293 | 0.0218 - 0.0287 |
| CH1S54 - LAGS58 | 0.0249 | 0.0246 | 0.0247 | 0.0244 | 0.0215 - 0.0299 | 0.0215 - 0.0291 |
| CH1S54 - VGS51 | 0.0249 | 0.0246 | 0.0247 | 0.0245 | 0.0222 - 0.0289 | 0.0222 - 0.0274 |
| CH1S54 - VGS53 | 0.0262 | 0.026 | 0.0261 | 0.026 | 0.0233 - 0.0302 | 0.0233 - 0.0290 |
| CH2S52 - CONM10 | 0.0254 | 0.0253 | 0.0259 | 0.0258 | 0.0180 - 0.0287 | 0.0180 - 0.0287 |
| CONS51 - CH1S54 | 0.0269 | 0.0259 | 0.0264 | 0.026 | 0.0233 - 0.0332 | 0.0233 - 0.0282 |
| CONS51 - CH2S52 | 0.0228 | 0.0221 | 0.0224 | 0.0221 | 0.0181 - 0.0285 | 0.0181 - 0.0250 |
| CONS51 - CONM10 | 0.0229 | 0.022 | 0.023 | 0.0224 | 0.0168 - 0.0302 | 0.0168 - 0.0249 |
| CONS51 - CONS52 | 0.0202 | 0.0196 | 0.02 | 0.0194 | 0.0167 - 0.0262 | 0.0167 - 0.0231 |
| CONS51 - LAGS53 | 0.0202 | 0.0194 | 0.0198 | 0.0192 | 0.0168 - 0.0268 | 0.0168 - 0.0229 |
| CONS51 - LAGS58 | 0.0198 | 0.0191 | 0.0193 | 0.0191 | 0.0159 - 0.0252 | 0.0159 - 0.0233 |
| CONS51 - VGS51 | 0.0211 | 0.0201 | 0.0207 | 0.0206 | 0.0182 - 0.0290 | 0.0182 - 0.0221 |
| CONS51 - VGS53 | 0.0225 | 0.0216 | 0.0218 | 0.0214 | 0.0184 - 0.0305 | 0.0184 - 0.0259 |
| CONS52 - CH1S54 | 0.0272 | 0.0268 | 0.0272 | 0.0268 | 0.0240 - 0.0307 | 0.0240 - 0.0302 |
| CONS52 - CH2S52 | 0.0227 | 0.0226 | 0.0235 | 0.0234 | 0.0074 - 0.0264 | 0.0087 - 0.0260 |
| CONS52 - CONM10 | 0.0228 | 0.0225 | 0.0231 | 0.0229 | 0.0170 - 0.0269 | 0.0170 - 0.0269 |
| CONS52 - LAGS53 | 0.0191 | 0.0189 | 0.0189 | 0.0187 | 0.0164 - 0.0227 | 0.0164 - 0.0227 |
| CONS52 - LAGS58 | 0.0192 | 0.0189 | 0.02 | 0.0197 | 0.0047 - 0.0230 | 0.0072 - 0.0230 |
| CONS52 - VGS51 | 0.0209 | 0.0207 | 0.0203 | 0.0203 | 0.0186 - 0.0236 | 0.0186 - 0.0236 |
| CONS52 - VGS53 | 0.0212 | 0.021 | 0.0209 | 0.0206 | 0.0184 - 0.0262 | 0.0184 - 0.0262 |
| LAGS53 - CH2S52 | 0.0223 | 0.0222 | 0.0224 | 0.0223 | 0.0176 - 0.0261 | 0.0176 - 0.0261 |
| LAGS53 - CONM10 | 0.0218 | 0.0215 | 0.0218 | 0.0216 | 0.0162 - 0.0261 | 0.0162 - 0.0261 |
| LAGS58 - CH2S52 | 0.022 | 0.0219 | 0.0218 | 0.0217 | 0.0095 - 0.0254 | 0.0095 - 0.0254 |
| LAGS58 - CONM10 | 0.0223 | 0.0221 | 0.0223 | 0.022 | 0.0160 - 0.0263 | 0.0160 - 0.0263 |
| LAGS58 - LAGS53 | 0.0172 | 0.017 | 0.0171 | 0.0169 | 0.0142 - 0.0212 | 0.0142 - 0.0212 |
| VGS51 - CH2S52 | 0.0206 | 0.0206 | 0.0207 | 0.0207 | 0.0185 - 0.0218 | 0.0185 - 0.0218 |
| VGS51 - CONM10 | 0.0224 | 0.0223 | 0.0224 | 0.0222 | 0.0177 - 0.0278 | 0.0177 - 0.0278 |
| VGS51 - LAGS53 | 0.0188 | 0.0186 | 0.0185 | 0.0184 | 0.0159 - 0.0229 | 0.0159 - 0.0229 |
| VGS51 - LAGS58 | 0.0184 | 0.0183 | 0.0181 | 0.0176 | 0.0157 - 0.0217 | 0.0157 - 0.0217 |
| VGS51 - VGS53 | 0.0165 | 0.0165 | 0.0162 | 0.0162 | 0.0140 - 0.0208 | 0.0140 - 0.0208 |
| VGS53 - CH2S52 | 0.0212 | 0.0212 | 0.0208 | 0.0208 | 0.0187 - 0.0252 | 0.0187 - 0.0252 |
| VGS53 - CONM10 | 0.0241 | 0.024 | 0.0245 | 0.0245 | 0.0182 - 0.0287 | 0.0182 - 0.0287 |
| VGS53 - LAGS53 | 0.0208 | 0.0207 | 0.0205 | 0.0204 | 0.0174 - 0.0263 | 0.0174 - 0.0263 |
| VGS53 - LAGS58 | 0.0202 | 0.0201 | 0.0199 | 0.0197 | 0.0169 - 0.0254 | 0.0169 - 0.0254 |

**Table S2**. Descriptive statistics of volatiles organic compounds (VOCs) emitted by wild cotton (*Gossypium hirsutum*) plants under control (undamaged) and herbivore-damaged treatments (damage by *Alabama argillacea*). Means and standard errors (ng h^-1^) are shown for each individual VOCs. Volatile collection was performed for 2 hours (see Methods). Asterisks indicate compounds identified previously via comparison to authentic standards in Mamin et al. 2025. The rest of the compounds were tentatively identified by mass spectra and retention time. RT= Retention Time. Bold compounds indicate that relative emission rates are substantially increased as a result of damage.

| **Volatile compound** | **RT** | **Control** | **Damaged** |
| --- | --- | --- | --- |
| Unknown aldoxime | 5.03 | 1.54 ± 0.13 | 2.64 ± 0.22 |
| (*Z*)-3-Hexenal | 5.53 | 3.39 ± .87 | 4.59 ± 1.01 |
| (*Z*)-3-Hexenol * | 6.96 | 9.63 ± 3.45 | 10.48± 2.65 |
| (*E*)-2-Methylbutyraldoxime * | 7.00 | 1.31 ± 0.32 | 2.93 ± 0.66 |
| (*Z*)-2-Methylbutyraldoxime * | 7.21 | 1.05 ± 0.15 | 1.06 ± 0.14 |
| 2-Methylbutyl acetate | 7.54 | 3.80 ± 0.80 | 11.57 ± 2.16 |
| α-Pinene * | 8.83 | 120.06 ± 29.40 | 156.54 ± 34.55 |
| Camphene | 9.19 | 9.89 ± 2.75 | 9.98 ± 2.74 |
| β-Pinene * | 9.86 | 30.14 ± 5.96 | 31.52 ±6.06 |
| 6-Methyl-5-hepten-2-one | 10.16 | 3.41 ± 0.74 | 6.75 ± 1.35 |
| β-Myrcene * | 10.21 | 22.43 ± 4.95 | 36.22 ± 7.59 |
| **(*Z*)-3-Hexenyl acetate *** | 10.59 | 49.99 ± 12.53 | 107.35 ± 22.59 |
| p-Cymene | 10.96 | 1.49 ± 0.90 | 2.17 ± 1.35 |
| Limonene * | 11.05 | 9.11 ± 1.47 | 10.20 ± 1.54 |
| (*Z*)-β-Ocimene | 11.24 | 2.23 ± .48 | 15.73 ± 3.20 |
| **(*E*)-β-Ocimene *** | 11.478 | 11.84 ± 1.53 | 2320.48 ± 29.83 |
| γ-Terpinene * | 11.69 | 1.24 ± 0.74 | 1.31 ± 0.75 |
| (*Z*)-Linalool loxide | 12.05 | 1.68 ± 0.31 | 5.07 ± 0.80 |
| (*E*)-Linalool oxide | 12.41 | 0.93 ± 0.30 | 1.21 ± 0.38 |
| Methyl benzoate * | 12.55 | 0.64 ± 0.16 | 10.58 ± 2.39 |
| Linalool * | 12.89 | 0.18 ± 0.04 | 0.46 ± 0.10 |
| **(*E*)-DMNT *** | 13.01 | 15.40 ± 3.38 | 132.13 ± 24.54 |
| Unknown monoterpene | 13.30 | 0.97 ± 0.25 | 2.03 ± 0.50 |
| Benzyl nitrile | 13.56 | 4.85 ± 0.50 | 5.65 ± 0.55 |
| (*E*)-Myroxide | 13.61 | 0.69 ± 0.13 | 1.68 ± 0.29 |
| (*E*)-3-Hexenyl butyrate | 13.63 | 0.55 ± 0.05 | 1.10 ± 0.10 |
| Unknown nitrile | 14.43 | 1.58 ± 0.16 | 3.97 ± 0.42 |
| Unknown nitrile | 14.57 | 3.30 ± 0.82 | 11.22 ± 2.58 |
| (*E*)-2-Hexenyl butyrate | 14.63 | 0.24 ± 0.05 | 3.29 ± 0.61 |
| Methyl salicylate * | 14.88 | 1.33 ± 0.14 | 3.75 ± 0.39 |
| (*Z*)-3-Hexenyl -α-methylbutyrate | 15.75 | 0.46 ± 0.09 | 9.59 ± 1.77 |
| **Indole *** | 17.23 | 1.08 ± 0.25 | 44.47 ± 8.16 |
| Unknown sesquiterpene | 19.24 | 1.34 ± 0.32 | 1.49 ± 0.34 |
| α-Copaene * | 19.31 | 0.73 ± 0.14 | 1.58 ± 0.29 |
| (*E*)-β-Caryophyllene * | 20.39 | 1.30 ± 0.48 | 2.71 ± 0.96 |
| **(*E*)-β-Farnesene *** | 21.14 | 2.08 ± 0.47 | 24.34 ± 5.21 |
| α-Humulene * | 21.20 | 1.08 ± 0.13 | 2.47 ± 0.31 |
| Bicyclogermacrene | 22.21 | 6.55 ± 0.59 | 8.71 ± 0.72 |
| (*E*, *E*)-α-Farnesene | 22.35 | 0.65 ± 0.11 | 2.33 ± 0.37 |

**Table S3**. Results from models testing for the effects of leaf damage treatment on the emission of different volatiles organic compounds (VOCs) from emitter cotton (*Gossypium hirsutum*) plants. We used linear mixed effects models (LMM) or generalized linear mixed models (GLMM) depending on the best distribution for the errors. We included plant height as a covariate (results not shown). F or Wald χ^2^ tests were used for LMMs and GLMMs, respectively. Statistically significant P-values are in bold text (*P* < 0.05).

|  | **Herbivory treatment** | | | |
| --- | --- | --- | --- | --- |
| **Volatile compound** | | ***F* or *χ²*** | **df** | **P** |
| β-Ocimene | | 106.59 | 1 | **<0.0001** |
| (*E*)-DMNT | | 74.15 | 1 | **<0.0001** |
| (*E*)-β-farnesene | | 81.48 | 1 | **<0.0001** |
| Indole | | 184.97 | 1 | **<0.0001** |
| α-Pinene | | 1.46 | 1, 21 | 0.369 |
| β-Pinene | | 0.09 | 1 | 0.872 |
| y-Terpinene | | 0.01 | 1 | 0.892 |
| (*E*)-β-Caryophyllene | | 7.30 | 1 | **0.011** |

**Table S4**. List of ant species recruiting to wild cotton (*Gossypium hirsutum*) plants during Experiment 1 (see *Methods*).

| **Ant species** | **Count** | **Percent of total** |
| --- | --- | --- |
| *Brachyomyrmex australis* | 855 | 58.48 |
| *Camponotus planatus* | 2 | 0.14 |
| *Crematogaster torosa* | 2 | 0.14 |
| *Forelius pruinosus* | 249 | 17.03 |
| *Monomorium ebeninum* | 16 | 1.09 |
| *Nylanderia steinheili* | 4 | 0.27 |
| *Pheidole* sp*.* | 167 | 11.42 |
| *Pseudomyrmex gracilis* | 1 | 0.07 |
| *Pseudomyrmex pallidus* | 1 | 0.07 |
| *Solenopsis geminata* | 165 | 11.29 |

**Table S5.** Results from a linear mixed effects model testing for effects of emitter herbivory (damaged vs. undamaged, fixed), emitter-receiver relatedness (same vs. different mother plant, fixed), and their interaction on metabolites such as gossypol and heliocides from leaves collected during Experiment 2. Shown are test statistics (*F* values), degrees of freedom, and significance values for each effect.

|  | **Gossypol** | | | **Heliocides** | | |  |
| --- | --- | --- | --- | --- | --- | --- | --- |
| **Effect** | ***F*** | **df** | ***P*** | ***F*** | **df** | ***P*** | |
| Emitter herbivory (H) | 2.12 | 1, 21 | 0.15 | 1.68 | 1, 20 | 0.20 | |
| Relatedness (R) | 1.11 | 1, 17 | 0.30 | 0.06 | 1, 18 | 0.80 | |
| H*R | 3.46 | 1, 24 | 0.07 | 3.21 | 1, 24 | 0.08 | |


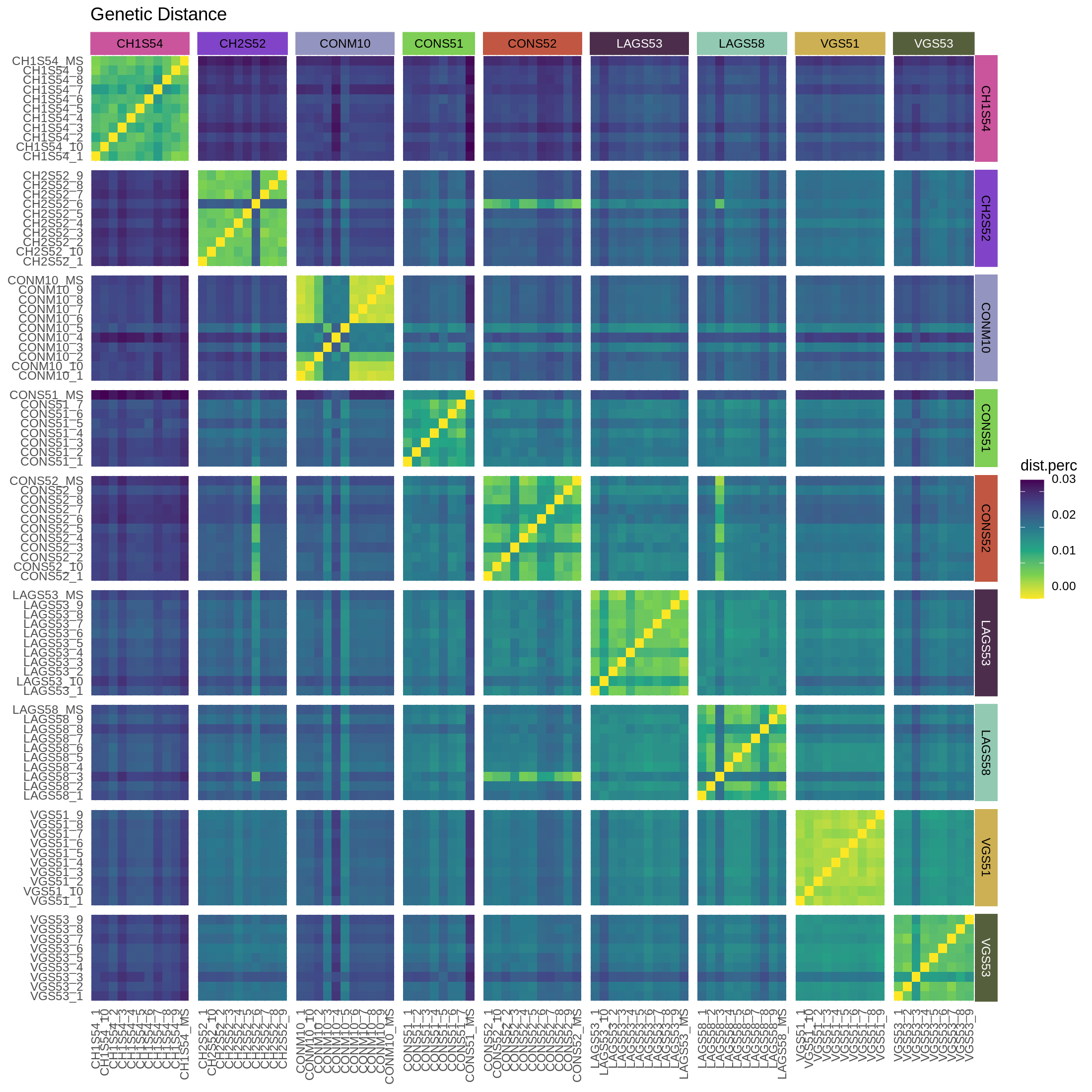


**Figure S1**. Genetic distance between and among families. Plants from the same source (i.e., mother plant + offspring) are grouped by site designation (e.g., CH1S54). Maternal plants (available in most but not all cases) are indicated by “_MS” and are listed first among the plants along the y-axis, and offspring are numbered. Within family pairwise genetic distance (see Table S1) varied from those with highly similar siblings (e.g., VGS51, mean distance: 0.0046) to those with more diverse siblings (e.g., CONS51, mean distance: 0.014). Likewise, interfamily pairwise genetics distances also varied from high similarity (e.g., mean distance: 0.0165 in VGS51 versus VGS53) to greater diversity (e.g., mean distance: 0.0282 in CH1S54 to CH2S52). These data were obtained from a subsample of plants (and mother plants) used in the experiments.

a)


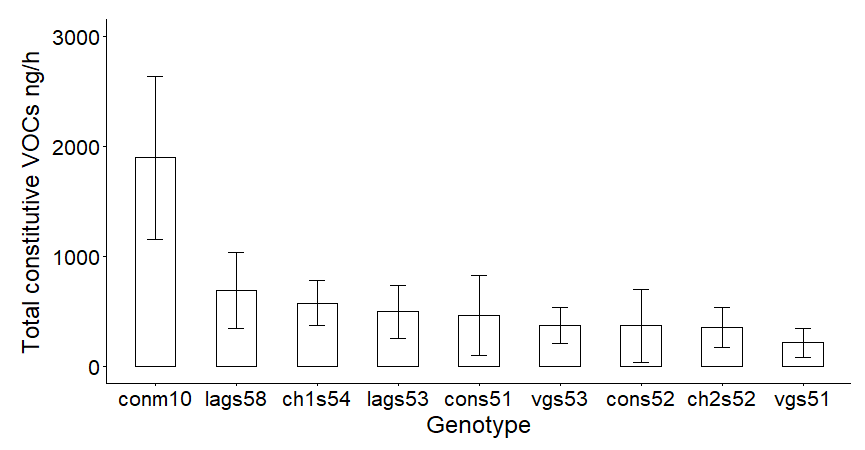

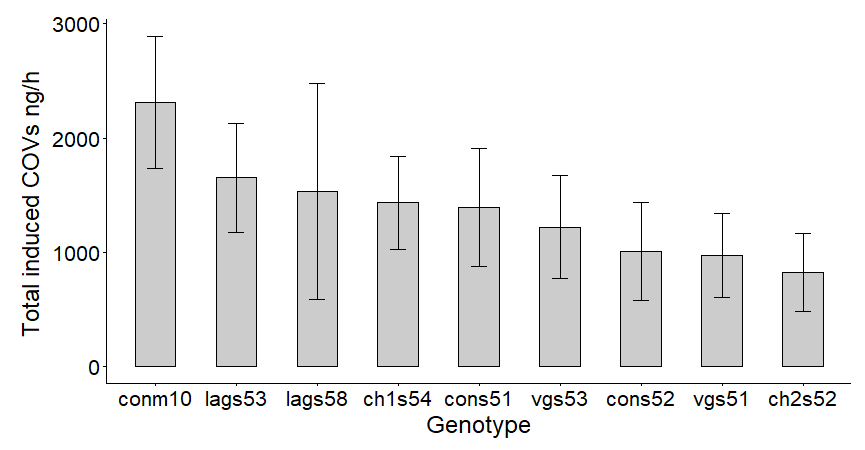


b)

Undamaged

Damaged

**Figure S2**. Total VOC emissions (ng h^-1^) across wild cotton (*Gossypium hirsutum*) mother plants (referred to as genotypes) for control (A) and damaged (B) plants. Bars are least squares means and standard errors from a linear mixed effects model (see *Methods*).


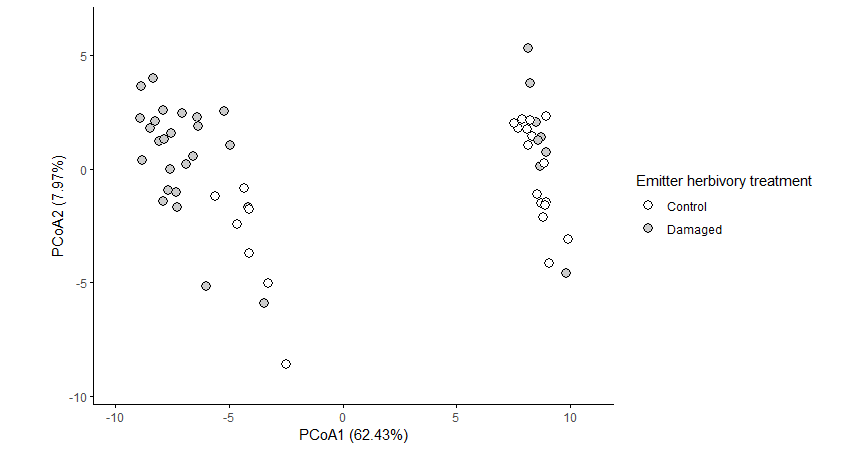


**Figure S3**. Effect of the emitter herbivory treatment on compositional variation in VOCs emissions based on a Principal Coordinates Analysis (PCoA) using compound relative abundances for control and damaged wild cotton (*Gossypium* *hirsutum*) plants. The first two axes together explained 70.04% of compositional variation in volatile emissions.

**Figure S4**. Variation in VOCs compositions among siblings of each mother plant based on a Principal Coordinates Analysis (PCoA) using compound relative abundances, shown separately for control (A) and damaged (B) wild cotton (*Gossypium* *hirsutum*) emitter plants. The first two axes together explained 70.04% of compositional variation in volatile emissions among mother plants.


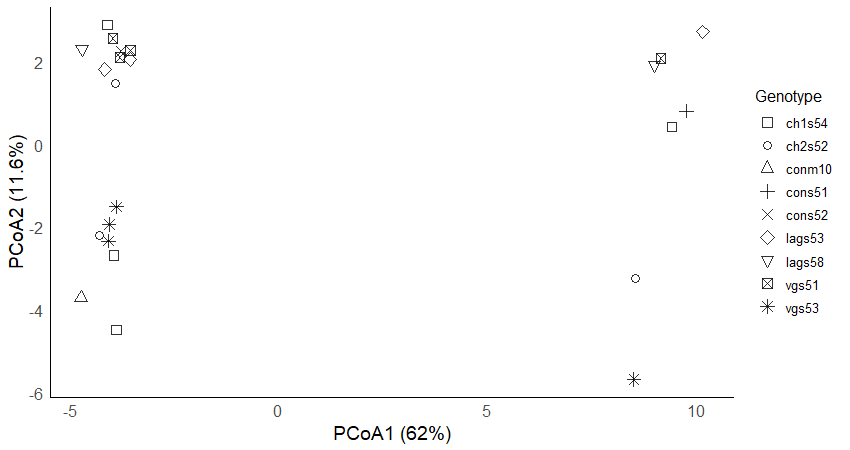


a)


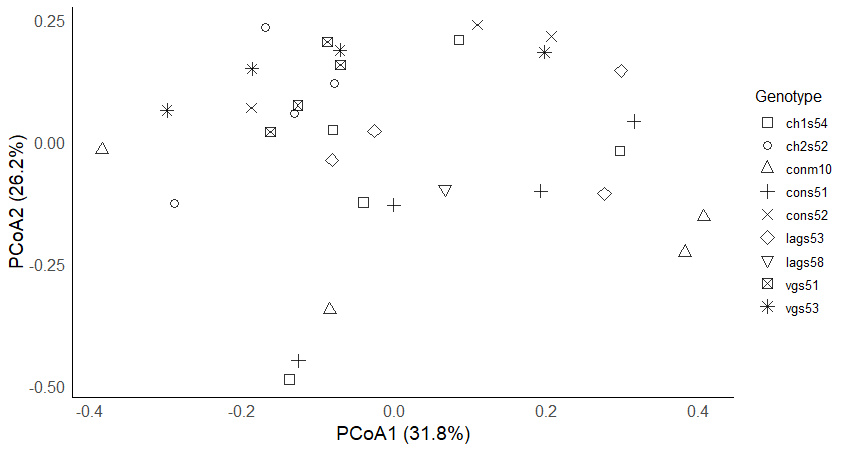


b)


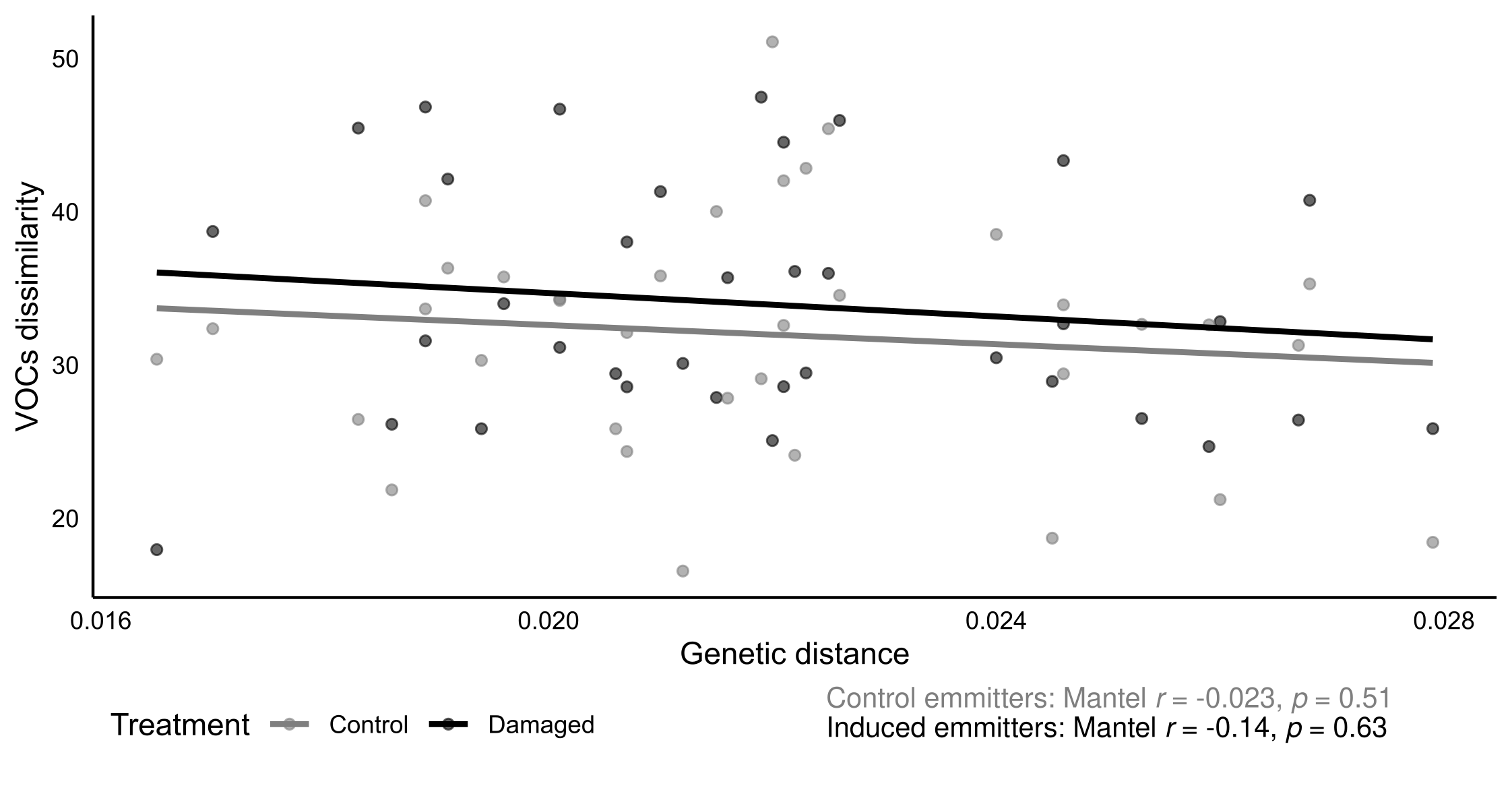


**Figure S5**. Correlation (Mantel tests) between genetic distance and VOC compositional dissimilarity across mother wild cotton plants, run separately for control (gray points and line) and induced (black points and line) plants (emitters).

**Literature cited**

Knaus, Brian J., and Niklaus J. Grünwald. 2017. “Vcfr: A Package to Manipulate and Visualize Variant Call Format Data in R.” *Molecular Ecology Resources* 17: 44–53. https://doi.org/10.1111/1755-0998.12549.

Li, Heng. 2013. “Aligning Sequence Reads, Clone Sequences and Assembly Contigs with BWA-MEM.” *ArXiv* 1303. https://doi.org/10.48550/arXiv.1303.3997.

Magbanua, Zenaida V., Chuan-Yu Hsu, Olga Pechanova, Mark Arick, Corrinne E. Grover, and Daniel G. Peterson. 2023. “Innovations in Double Digest Restriction-Site Associated DNA Sequencing (ddRAD-Seq) Method for More Efficient SNP Identification.” *Analytical Biochemistry* 662: 115001. <https://doi.org/10.1016/j.ab.2022.115001>.

Mamin M, Clancy MV, Flückiger G, et al. 2025. Induction by caterpillars of stored and emitted volatiles in terpene chemotypes from populations of wild cotton (*Gossypium hirsutum*). *BMC Plant Biology* 25: 127. https://doi.org/10.1186/s12870-025-06088-7

Martin, Marcel. 2011. “Cutadapt Removes Adapter Sequences from High-Throughput Sequencing Reads.” *EMBnet.Journal* 17: 10–12. https://doi.org/10.14806/ej.17.1.200.

Ning, Weixuan, Karen M. Rogers, Chuan-Yu Hsu, Zenaida V. Magbanua, Olga Pechanova, Mark A. Arick, Ehsan Kayal, et al. 2024. “Origin and Diversity of the Wild Cottons (*Gossypium hirsutum*) of Mound Key, Florida.” *Scientific Reports* 14: 14046. https://doi.org/10.1038/s41598-024-64887-8.

Paradis, Emmanuel, and Klaus Schliep. 2019. “Ape 5.0: An Environment for Modern Phylogenetics and Evolutionary Analyses in R.” *Bioinformatics* 35: 526–28. https://doi.org/10.1093/bioinformatics/bty633.

R Core Team. 2024. “R: A Language and Environment for Statistical Computing.” Vienna, Austria: R Foundation for Statistical Computing. https://www.R-project.org/.

Rochette, Nicolas C., Angel G. Rivera-Colón, and Julian M. Catchen. 2019. “Stacks 2: Analytical Methods for Paired-End Sequencing Improve RADseq-Based Population Genomics.” *Molecular Ecology* 28: 4737–54. https://doi.org/10.1111/mec.15253.

Rognes, Torbjørn, Tomáš Flouri, Ben Nichols, Christopher Quince, and Frédéric Mahé. 2016. “VSEARCH: A Versatile Open Source Tool for Metagenomics.” Edited by Tomas Hrbek. *PeerJ* 4: e2584. https://doi.org/10.7717/peerj.2584.

Wickham, Hadley. 2016. *Ggplot2: Elegant Graphics for Data Analysis*. Springer-Verlag New York. https://ggplot2.tidyverse.org.

Zheng, Xiuwen, David Levine, Jess Shen, Stephanie Gogarten, Cathy Laurie, and Bruce Weir. 2012. “A High-Performance Computing Toolset for Relatedness and Principal Component Analysis of SNP Data.” *Bioinformatics (Oxford, England)* 28. https://doi.org/10.1093/bioinformatics/bts606.
